# Boosting the biosynthesis of betulinic acid and related triterpenoids in *Yarrowia lipolytica* via multimodular metabolic engineering

**DOI:** 10.1186/s12934-019-1127-8

**Published:** 2019-05-03

**Authors:** Cong-Cong Jin, Jin-Lai Zhang, Hao Song, Ying-Xiu Cao

**Affiliations:** 1 Frontier Science Center for Synthetic Biology and Key Laboratory of Systems Bioengineering (Ministry of Education), Tianjin University, Tianjin, 300072 China; 2 Collaborative Innovation Center of Chemical Science and Engineering (Tianjin), School of Chemical Engineering and Technology, Tianjin University, Tianjin, 300072 China

**Keywords:** Betulinic acid, Triterpenoids, P450, Multimodular metabolic engineering, *Yarrowia lipolytica*

## Abstract

**Background:**

Betulinic acid is a pentacyclic lupane-type triterpenoid and a potential antiviral and antitumor drug, but the amount of betulinic acid in plants is low and cannot meet the demand for this compound. *Yarrowia lipolytica*, as an oleaginous yeast, is a promising microbial cell factory for the production of highly hydrophobic compounds due to the ability of this organism to accumulate large amounts of lipids that can store hydrophobic products and supply sufficient precursors for terpene synthesis. However, engineering for the heterologous production of betulinic acid and related triterpenoids has not developed as systematically as that for the production of other terpenoids, thus the production of betulinic acid in microbes remains unsatisfactory.

**Results:**

In this study, we applied a multimodular strategy to systematically improve the biosynthesis of betulinic acid and related triterpenoids in *Y. lipolytica* by engineering four functional modules, namely, the heterogenous CYP/CPR, MVA, acetyl-CoA generation, and redox cofactor supply modules. First, by screening 25 combinations of cytochrome P450 monooxygenases (CYPs) and NADPH-cytochrome P450 reductases (CPRs), each of which originated from 5 different sources, we selected two optimal betulinic acid-producing strains. Then, *ERG1*, *ERG9*, and *HMG1* in the MVA module were overexpressed in the two strains, which dramatically increased betulinic acid production and resulted in a strain (YLJCC56) that exhibited the highest betulinic acid yield of 51.87 ± 2.77 mg/L. Then, we engineered the redox cofactor supply module by introducing NADPH- or NADH-generating enzymes and the acetyl-CoA generation module by directly overexpressing acetyl-CoA synthases or reinforcing the β-oxidation pathway, which further increased the total triterpenoid yield (the sum of the betulin, betulinic acid, betulinic aldehyde yields). Finally, we engineered these modules in combination, and the total triterpenoid yield reached 204.89 ± 11.56 mg/L (composed of 65.44% betulin, 23.71% betulinic acid and 10.85% betulinic aldehyde) in shake flask cultures.

**Conclusions:**

Here, we systematically engineered *Y. lipolytica* and achieved, to the best of our knowledge, the highest betulinic acid and total triterpenoid yields reported in microbes. Our study provides a suitable reference for studies on heterologous exploitation of P450 enzymes and manipulation of triterpenoid production in *Y. lipolytica*.

**Electronic supplementary material:**

The online version of this article (10.1186/s12934-019-1127-8) contains supplementary material, which is available to authorized users.

## Background

Betulinic acid is a pentacyclic lupane-type triterpenoid that widely exists in plants and possesses many pharmacological activities, such as antiviral (especially anti-HIV infection) [1, 2], antitumor (as a melanoma-specific cytotoxic agent) [3, 4] and other antimetabolic syndrome activities [5–8]. This compound can be acquired by purification of extracts from the outer bark of a variety of tree species [9]. Though this process has been optimized, problems such as low natural abundance, requirement of a large amount of land resources and long processing time are nonnegligible. The necessity for the development of an economical, ecofriendly and efficient production process has inspired researchers to use microorganisms for the production of betulinic acid [10].

**Fig. 1:**
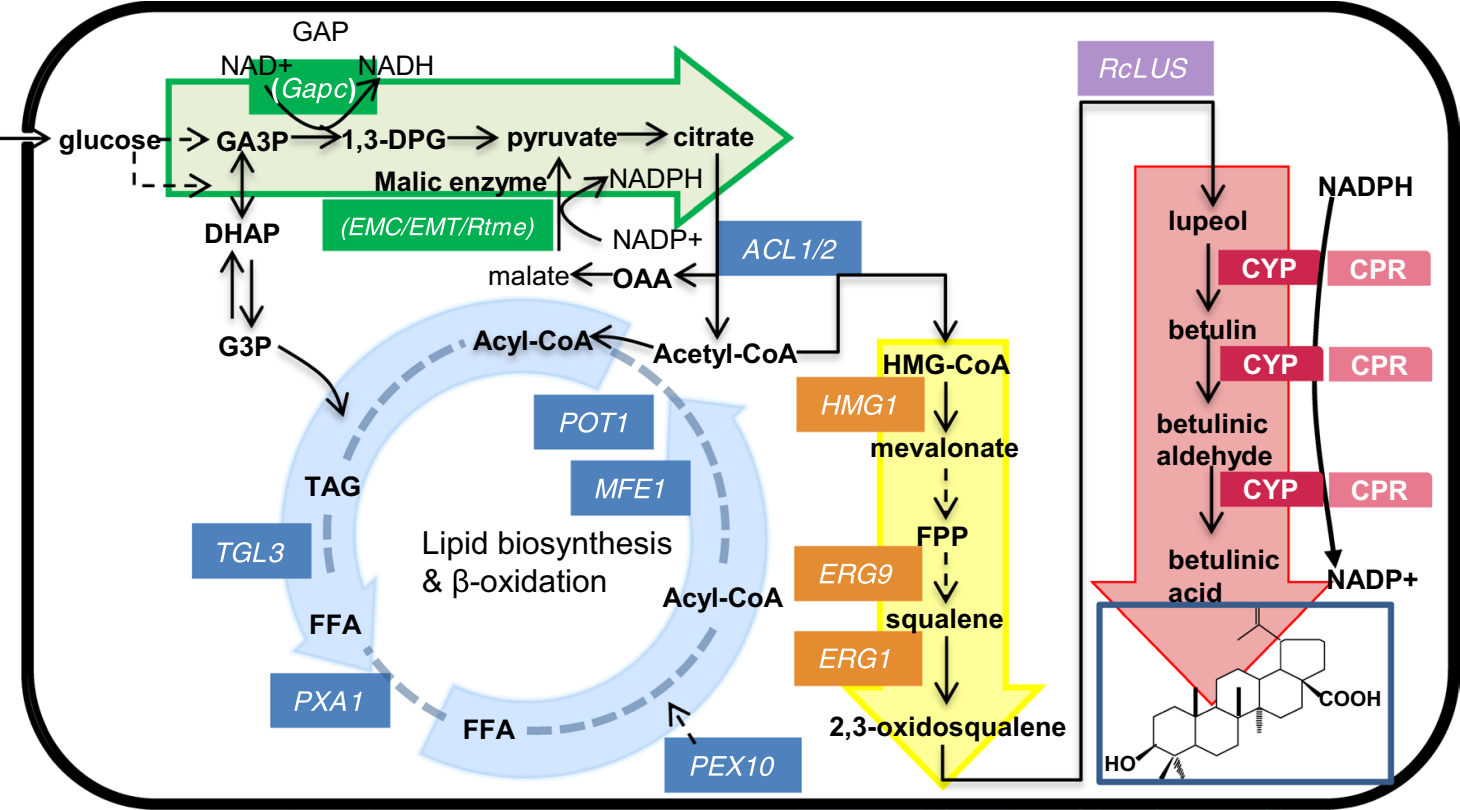
Overview of the multimodular strategy for betulinic acid-related triterpenoid production in *Y. lipolytica*. All the genes that were engineered in this study are presented, and the betulinic acid biosynthesis pathway was divided into four modules: the red arrow represents the heterogenous CYP/CPR module (CYP is lupeol C-28 oxidase and CPR is NADPH-cytochrome P450 reductase. Both originate from the five sources). The yellow arrow represents the MVA module with 3 genes (*ERG1/ERG9/HMG1*) that are overexpressed separately or simultaneously. The green arrow represents the redox cofactor supply module with four introduced genes, namely, *EMC, EMT, Rtme* (encoding malic enzyme, which is responsible for NADPH generation) and *Gapc* (encoding glyceraldehyde-3-phosphate dehydrogenase, which is responsible for NADH generation). The blue arrow represents the acetyl-CoA generation module with seven endogenous genes overexpressed (*ACL1* and *ACL2*, encoding ATP citrate lyase, which can directly increase acetyl-CoA levels; *PXA1*, *MFE1*, *PEX10*, *POT1* and *TGL3* in the β-oxidation pathway, which are responsible for the catabolism of fatty acids to generate acetyl-CoA)

The biosynthetic pathway of betulinic acid from glucose in *Yarrowia lipolytica* is shown in Fig. 1. In yeast, the biosynthetic precursors of betulinic acid are isopentenyl pyrophosphate (IPP) and dimethylallyl pyrophosphate (DMAPP), which are derived from acetyl-CoA via the mevalonic acid (MVA) pathway [11–13]. IPP then gives rise to higher order building blocks, namely, geranyl pyrophosphate (GPP) and farnesyl pyrophosphate (FPP), via the action of prenyltransferase enzymes. Two FPPs are condensed into squalene via a reaction catalyzed by squalene synthase (encoded by *ERG9*) and then converted to 2,3-oxidosqualene via a reaction catalyzed by squalene epoxidase (encoded by *ERG1*). The first step in the exogenous biosynthesis of betulinic acid from endogenous 2,3-oxidosqualene is catalyzed by lupeol synthase (LUS), which is followed by three sequential oxidations catalyzed by lupeol C-28 oxidases (cytochrome P450 monooxygenases, CYPs), leading to the generation of betulin, betulinic aldehyde, and betulinic acid. As increasing CYPs have been mined by bioinformatics analysis [14, 15], amino acid sequence alignment [16–18], and the combination approach of activation tagging and reverse genetic TILLING [19], heterologous betulinic acid production has been established in microbes [16, 20–23]. Li et al. demonstrated that attenuation of the competitive fatty acid biosynthesis pathway [23] and increasing the NADPH and oxygen supply [21] were beneficial for improvement of the production capability of betulinic acid in *Saccharomyces cerevisiae*. The titer of betulinic acid in yeast could be enhanced to 0.16 mg/L/OD_600_ by applying a multiple-approach strategy, which included using a novel CYP, employing a better yeast strain and upregulating the expression of key genes in the betulinic acid biosynthesis pathway [16]. Moreover, multicopy integration of *AtLUP1* and *CYP716Al1* with single-copy integration of *ATR2* and overexpression of endogenous *ERG1* and *tHMG1* could improve betulinic acid production to ~ 28 mg/L in shake flask cultures in *S. cerevisiae* [22]. Furthermore, by coexpression of LUS and the fusion protein of CYP with CPR and multicopy integration of *tHMG1* and *ERG9*, betulinic acid biosynthesis was achieved in *Y. lipolytica* and the yield was improved to 26.53 mg/L by substitution of glycerol for glucose as a carbon source [20]. Although the exogenous production pathway of betulinic acid has been introduced in yeast, no considerable yield was observed, and the engineering of heterologous production of betulinic acid was not performed as systematically as that of the production of other terpenoids.

Multimodular metabolic engineering is an efficient strategy for addressing perturbances such as imbalance in pathway flux, module incompatibility or accumulation of intermediates caused by manipulation of endogenous genes and the introduction of heterologous pathways, and this method enables systematic manipulation of strains and metabolic pathways [24, 25]. This potential strategy has been widely implemented in *Escherichia coli* and *S. cerevisiae* for the production of value-added compounds. By partitioning the biosynthetic pathway of the diterpene taxadiene into two modules—an upstream methylerythritol-phosphate (MEP) module and a heterologous downstream terpenoid-forming module—and balancing the two modules by using various promoters and gene copy numbers in *E. coli*, production of the taxadiene was increased 15,000-fold compared to that in the parent strain and a yield of 1.02 ± 0.08 g/L achieved in fed-batch bioreactor fermentations [26]. Similarly, the production of miltiradiene (a kind of diterpenoid) in *S. cerevisiae* was improved from 0.5 to 12.5 mg/L by engineering MVA and heterologous synthase modules [27]. Recently, this strategy was also used to boost the production of a kind of triterpene—protopanaxadiol. By dividing the biosynthetic pathway of this compound into four modules—MVA, triterpene biosynthesis, sterol biosynthesis, and acetyl-CoA formation modules—and manipulating these modules simultaneously, protopanaxadiol production was enhanced to 66.55 mg/L/OD_600_ [28]. These studies demonstrated the potential of multimodular metabolic engineering for high-level production of terpenes and for addressing module incompatibility.

*Yarrowia lipolytica*, as a GRAS (generally recognized as safe) and oleaginous microorganism, has become a potential platform for the production of highly hydrophobic compounds [29, 30] because of the ability of this organism to accumulate large amounts of lipids that can store hydrophobic products [31, 32] and to supply sufficient intermediates and energy for terpene synthesis [33, 34]. In addition, the recent development of efficient and predictable synthetic biological tools in *Y. lipolytica* has contributed to the convenience of genetic manipulation in this organism [35–38]. Based on these factors, *Y. lipolytica* has been used to produce various hydrophobic terpenoids, such as linalool (monoterpene alcohol) [39], α-farnesene (sesquiterpene) [40], squalene (triterpene) [41], β-carotene, astaxanthin and lycopene (tetraterpene) [42–45]. In some cases, terpenoid production in *Y. lipolytica* was even higher than that in *S. cerevisiae*. For example, maximum production of β-carotene in *Y. lipolytica* was realized at 6.5 g/L [43], which demonstrated the potential of this strain for terpenoid production.

In this study, we used a multimodular metabolic engineering strategy to systematically engineer *Y. lipolytica* for production of the hydrophobic betulinic acid and related triterpenoids by dividing the biosynthetic pathway into four functional modules, including the heterogenous CYP/CPR, MVA, acetyl-CoA generation, and redox cofactor supply modules. By introducing exogenous genes or overexpressing endogenous key genes associated with these modules, we obtained a 51.87 ± 2.77 mg/L yield of betulinic acid and a 204.89 ± 11.56 mg/L yield of triterpenoids, both of which were the highest yields reported in shake flask cultures.

## Results and discussion

### Screening the CYP/CPR module for biosynthesis of betulinic acid in *Y. lipolytica*

**Fig. 2:**
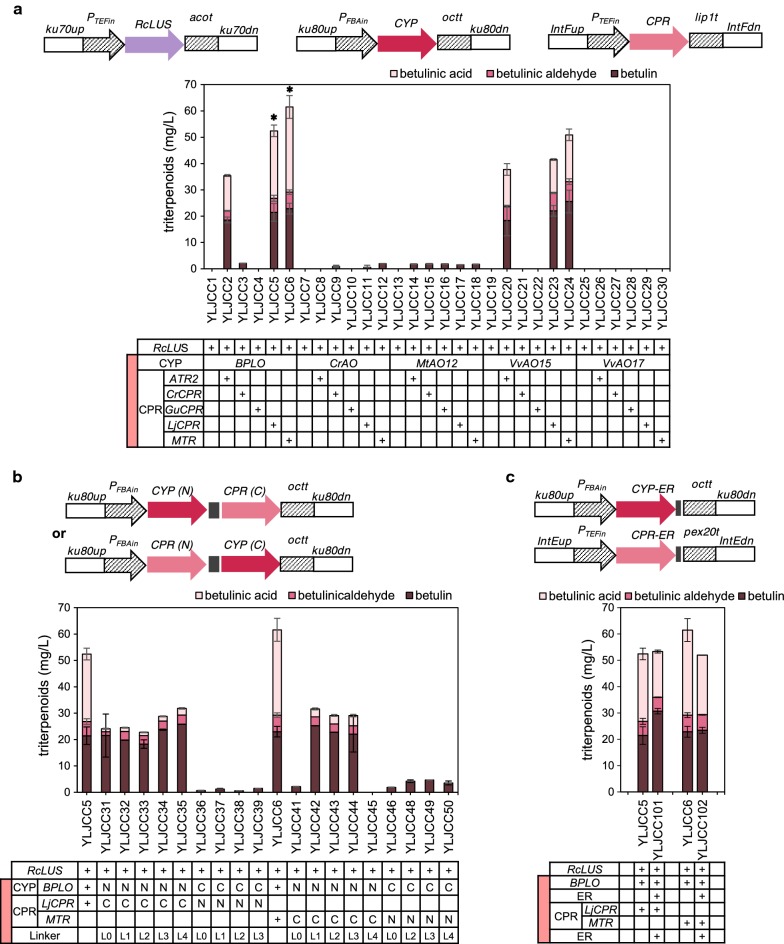
Screening of CYP/CPR for betulinic acid production. **a** Triterpenoid production by 30 strains that coexpressed LUS with CYP or LUS with CYP and CPR (both CYPs and CPRs originated from 5 different sources). **b** Triterpenoid production by strains that coexpressed LUS and fused CYP with CPR. N indicates that the encoded protein was located at the N-terminus of the fusion protein; C indicates that the encoded protein was located at the C-terminus of the fusion protein. L0 indicates that the two proteins were fused without any linker. L1, L2, L3 and L4 represent four different linkers, with amino acid sequences GGGS, GSG, GGGGS, and EAAAK, respectively. **c** Triterpenoid production of strains that coexpressed LUS and endoplasmic reticulum (ER)-targeted CYP and CPR. The ER-targeting sequence was fused to the C-terminus of both CYP and CPR. The red block in the first column of the gene forms represents the CYP/CPR module. Asterisk represents the strains used for subsequent optimization

Lupeol is the first heterologous intermediate metabolite from 2,3-oxidosqualene to betulinic acid, and this metabolite can be produced by the expression of LUSs. Subsequently, CYP716A subfamily members, which were identified as multifunctional triterpene C-28 oxidases [15], catalyze three sequential oxidations of lupeol to generate betulin, betulinic aldehyde and betulinic acid (Additional file 1: Figure S1). In this study, the *RcLUS* gene from *Ricinus communis* under the control of the TEFin promoter was integrated into the *Ku70* locus (YALI0C08701 g) of *Y. lipolytica* to produce lupeol (Fig. 2a, Additional file 2: Figure S2). Because the native P450 enzymes of *Y. lipolytica* did not catalyze betulinic acid production (Additional file 3: Table S1), we tried to screen effective CYPs from plant species. Five CYP genes, namely, *BPLO* (CYP716A180) from *Betula platyphylla* [16], *CrAO* (CYP716AL1) from *Catharanthus roseus* [12], *MtAO12* (CYP716A12) from *Medicago truncatula* [15], and *VvAO15* (CYP716A15) and *VvAO17* (CYP716A17) from *Vitis vinifera* [15], were expressed under the FBAin promoter and integrated into the *Ku80* locus (YALI0E02068g), which generated strains YLJCC1, YLJCC7, YLJCC13, YLJCC19, YLJCC25. However, the desired product was not detected (Fig. 2a).

Previous studies have shown that in *S. cerevisiae*, recombinant plant CYPs work well with endogenous CPRs [21, 23]. However, our results and those of several other studies [20, 46, 47] indicated that in *Y. lipolytica*, plant CYPs might require the coexpression of heterogenous CPRs for exogenous terpenoid production. Accordingly, five CPR genes from different sources, namely, *ATR2* from *Arabidopsis thaliana*, *CrCPR* from C*. roseus*, *GuCPR* from *Glycyrrhiza uralensis*, *LjCPR* from *Lotus japonicus* and *MTR* from *M. truncatula*, were expressed under the TEFin promoter and integrated into the IntF site (YALI0F3161413 to YALI0F3162449) [31]. Twenty-five strains were generated, and the fermentation results are shown in Fig. 2a (GC profiles and mass spectra of YLJCC6 are shown as an example in Additional file 4: Figure S3a and b). The results show that the combinations of CYPs from two sources (BPLO or VvAO15) and CPRs from three sources (ATR2, LjCPR, or MTR) could achieve the synthesis of betulinic acid (six strains were generated: YLJCC2, YLJCC5, YLJCC6, YLJCC20, YLJCC23, YLJCC24). Among the various CYPs, BPLO possesses relatively higher catalytic capacity for betulinic acid production in *S. cerevisiae* [16, 17]. In this study, BPLO also showed high catalytic capacity in *Y. lipolytica*. VvAO15 shared close evolutionary relationships and high homology with BPLO (Additional file 5: Figure S4a and b), which might be the reason why VvAO15 also performed well in *Y. lipolytica*. In addition, among the six strains, YLJCC5 and YLJCC6 showed the highest production of betulinic acid and total triterpenoids (Fig. 2a), which might be caused by the efficient interaction between BPLO and the related CPRs (that is LjCPR or MTR) [17, 48]. The titer of betulinic acid in YLJCC5 and YLJCC6 was 25.62 ± 2.20 mg/L and 32.33 ± 4.34 mg/L, respectively.

To further optimize the CYP/CPR module, we tried to fuse BPLO with LjCPR or MTR or to localize the proteins in the endoplasmic reticulum (ER) (Fig. 2b, c). We used two strategies for fusion of CYP and CPR. In the first strategy, the two proteins were fused without any linker and designated L0. In the second strategy, CYP and CPR were fused with four different linkers, designated L1, L2, L3 and L4. The amino acid sequences of these four linkers were GGGS, GSG, GGGGS, EAAAK, where the first three represent flexible linkers and the last one represents a rigid linker. The results showed that when CYP was fused to the N-terminus of the CPRs, the generated strains YLJCC31-35 and YLJCC41-45 showed a dramatic decline in the production of betulinic acid compared with the control strains, regardless of the strategy of fusion (with or without linker, or with any kind of linker). However, when CYP was fused to the C-terminus of the CPRs, the engineered strains barely produced any triterpenoids (Fig. 2b). In summary, the fusion of CYP with CPR could not further improve betulinic acid production. On the other hand, an ER-targeting peptide (KDEL) was fused to the C-terminus of the CYP (BPLO) and CPRs (LjCPR and MTR) [30] to generate strains YLJCC101 and YLJCC102, which coexpressed LUS, CYP-ER and CPR-ER (Fig. 2c). Although previous studies reported that the involvement of the ER could facilitate P450 function [49, 50], neither total triterpenoid production nor betulinic acid production was improved in the engineered strains, possibly because we used the full-length sequences of CYP and CPR, not truncated sequences, for heterologous expression, and these sequences already have signal peptides in the N-terminus of the proteins for membrane translocation [26, 51]. We fused a sfGFP [52] to the C-terminus of BPLO, LjCPR and MTR (without the ER-targeting peptide), and the confocal microscopy images confirmed our hypothesis. As shown in Additional file 6: Figure S5, the expressed proteins were already located in the membrane or organelle in *Y. lipolytica* due to the presence of the native signal peptide.

Finally, because no further improvement was observed upon fusion of CYP with CPR or upon addition of the ER-targeting peptide, the two best production strains from the above optimization, namely, YLJCC5 and YLJCC6 (Fig. 2a), were selected and subjected to further engineering.

### Improving the production of triterpenoids by engineering the MVA module

**Fig. 3:**
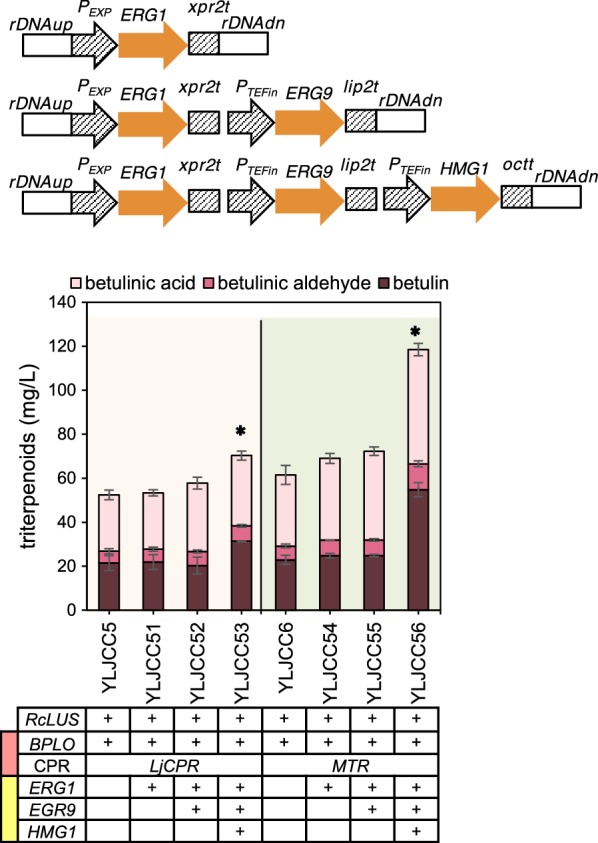
Engineering of the MVA module to enhance betulinic acid production. The genes *ERG1*, *ERG9* and *HMG1* in the MVA module were overexpressed individually or in combination. The light orange background represents strains generated from YLJCC5, and the reseda background represents strains generated from YLJCC6. Red and yellow blocks in the first column of the gene form represent the CYP/CPR and MVA modules, respectively. Asterisk represents the strains used in the final multimodular optimization

Although the betulinic acid heterologous biosynthetic module was constructed in *Y. lipolytica*, the quantities of betulinic acid produced by strains YLJCC5 and YLJCC6 remained low. To increase the supply of the 2,3-oxidosqualene precursor, we engineered the MVA module with three structural genes, namely, *ERG1*, *ERG9* and *HMG1*. These genes were overexpressed individually or in combination. Expression cassettes of these structural genes were integrated into the rDNA site of the *Y. lipolytica* genome (Fig. 3).

First, squalene epoxidase (encoded by *ERG1*), which is responsible for the first oxygenation step in sterol biosynthesis and is recognized as the key enzyme in this pathway [53], was overexpressed under the control of the EXP promoter in strains YLJCC5 and YLJCC6, generating strains YLJCC51 and YLJCC54. As shown in Fig. 3, the total triterpenoid levels in YLJCC51 and YLJCC54 were slightly increased. Then, we further overexpressed squalene synthase (encoded by *ERG9*), which converts FPP to squalene and represents the branching point of synthetic pathways of triterpenoids, sterols and sesquiterpenoids. The resulting strains, namely, YLJCC52 and YLJCC55, exhibited a further increase in betulinic acid and total triterpenoid production (Fig. 3). Finally, with the addition of *HMG1*, which encodes a rate-limiting enzyme in the MVA pathway, the production of betulinic acid in the generated strains YLJCC53 and YLJCC56 improved 1.25- and 1.60-fold, respectively, compared with YLJCC5 and YLJCC6, and the total triterpenoid production increased by 1.34-fold and 1.91-fold, respectively (Fig. 3).

Previously, overexpression of *ERG1* in *S. cerevisiae* increased the levels of the triterpenoid protopanaxadiol [54]. It has been demonstrated that overexpression of *ERG9* enhanced triterpenoid accumulation in plants [55–57] and β-amyrin production in *S. cerevisiae* [58]. Overexpression of *tHMG1* (encoding the N-terminal truncated version of HMG1) is a commonly used approach to improve the production of terpenoids because this method can overcome the tight regulation of *HMG1* in host cells [40, 42, 59–61]. However, some data indicated that overexpression of homologous *HMG1* was more effective than overexpression of *tHMG1* for production of the monoterpene limonene in *Y. lipolytica* [62]. Recently, overexpression of endogenous *HMG1* improved squalene synthesis 3.2-fold in *Y. lipolytica* [41]. Hence, we chose to overexpress *HMG1* rather than *tHMG1* in *Y. lipolytica*. Our results suggested that overexpression of *ERG1* and *ERG9* only slightly enhanced betulinic acid production in *Y. lipolytica.* Further overexpression of *HMG1* led to a marked improvement in the yield of the product.

### Improving triterpenoid production via engineering of the redox cofactor supply module

**Fig. 4:**
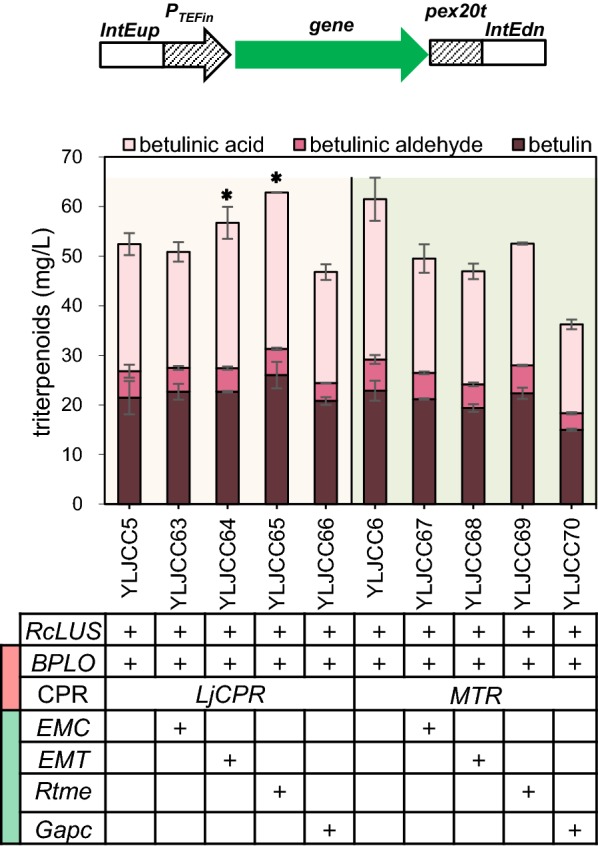
Engineering of the redox cofactor supply module to improve triterpenoid production. The *EMC*, *EMT* and *Rtme* genes that encode malic enzymes are responsible for NADPH generation. The gene *Gapc*, encoding glyceraldehyde-3-phosphate dehydrogenase, is responsible for NADH generation. The light orange background represents strains generated from YLJCC5, and the reseda background represents strains generated from YLJCC6. Red and green blocks in the first column of the gene form represent the CYP/CPR and redox cofactor supply modules, respectively. Asterisk represents the strains that exhibited increased triterpenoid production, and similar engineering was used in the final multimodular optimization

One of the key factors required for triterpenoid biosynthesis is sufficient redox supply [21]. Here, we tried to improve the NADPH or NADH supply by overexpression of malic enzyme or glyceraldehyde-3-phosphate (G3P) dehydrogenase, respectively. *EMC*, *EMT* and *Rtme* encoding malic enzyme and *Gapc* encoding G3P dehydrogenase (shown in Fig. 1) were integrated into the IntE site (YALI0E3673678–YALI0E3672531) [31] of YLJCC5 and YLJCC6 (Fig. 4), respectively, to generate strains YLJCC63-YLJCC70. The fermentation results are shown in Fig. 4. Compared with YLJCC5, the production of total triterpenoids and betulinic acid in YLJCC64 and YLJCC65 (harboring the introduced genes *EMT* and *Rtme*) increased slightly, although this increasing trend was not observed in YLJCC68 and YLJCC69 compared with YLJCC6. In contrast, when *Gapc*, which is responsible for NADH generation, was overexpressed, triterpenoid production decreased (YLJCC66). These results demonstrated that manipulation of the NADPH supply was more beneficial than manipulation of the NADH supply for betulinic acid production, which might be because NADPH is the direct redox supplier for endogenous MVA and the heterogenous CYP/CPR system. A previous study also demonstrated that improvement of the NADPH supply was an efficient method for improvement of betulinic acid production by introduction of *mBDH1,* which can increase cellular NADPH level with acetoin supplementation [21].

### Improving triterpenoid synthesis via engineering of the acetyl-CoA generation module

**Fig. 5:**
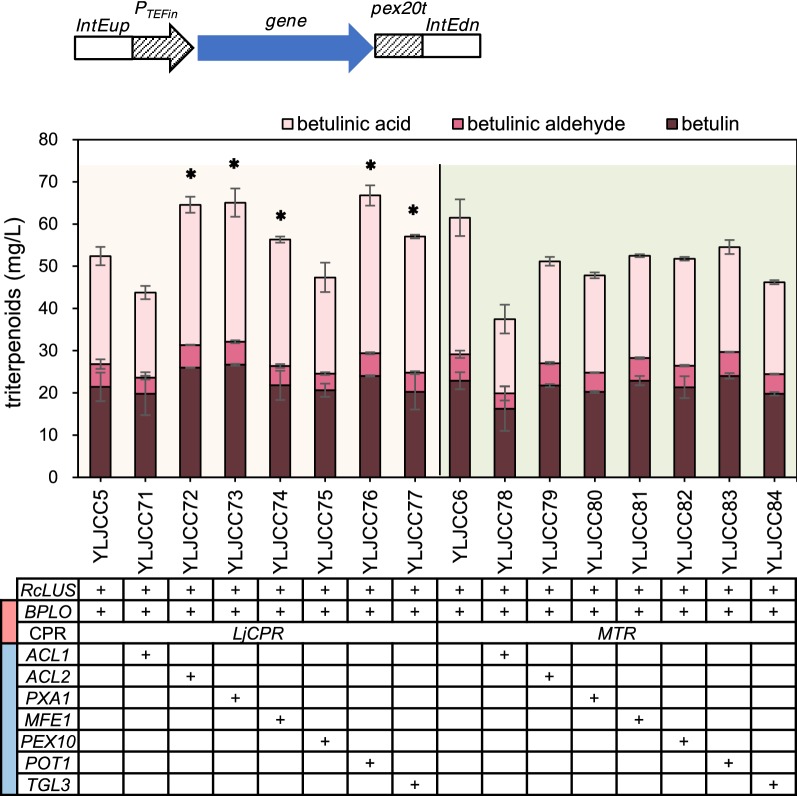
Engineering of the acetyl-CoA generation module to increase triterpenoid production. *ACL1* and *ACL2* encode ATP citrate lyase, which can directly increase acetyl-CoA levels; *PXA1*, *MFE1*, *PEX10*, *POT1*, and *TGL3* in the β-oxidation pathway are responsible for the catabolism of fatty acids to acetyl-CoA. The light orange background represents strains generated from YLJCC5, and the reseda background represents strains generated from YLJCC6. Red and blue blocks in the first column of gene form represent the CYP/CPR and acetyl-CoA generation modules, respectively. Asterisk represents the strains that exhibited increased triterpenoid production, and similar engineering was used in the final multimodular optimization

Acetyl-CoA is the biosynthetic precursor of lipids, terpenes and sterols. *Y. lipolytica,* which has been characterized as a nonconventional yeast, is able to accumulate large amounts of intracellular lipids, which seems to be a primary competition pathway for terpene production. To increase the carbon flux into the MVA pathway, we tried to recover acetyl-CoA from lipids by enhancing β-oxidation of fatty acids via overexpression of four structural genes, namely, the triacylglycerol lipase gene (*TGL3*), long-chain fatty acid transporter gene (*PXA1*), multifunctional β-oxidation enzyme gene (*MFE1*), 3-ketoacyl-CoA thiolase gene (*POT1*), and one regulatory gene, namely, the regulatory gene *PEX10*, which is necessary for correct peroxisomal biogenesis and morphology for the β-oxidation process (shown in Fig. 1). Moreover, we also overexpressed ATP citrate lyase (*ACL1* and *ACL2*) to directly increase the synthesis of acetyl-CoA. In summary, the engineered strains YLJCC71-YLJCC84 were constructed, and the fermentation results are shown in Fig. 5.

Overexpression of the endogenous ATP citrate lyase gene (*ylACL1*) has been previously shown to increase the level of cytosolic acetyl-CoA 1.5-fold in *Y. lipolytica* [41, 63]. However, in this study, overexpression of *ACL1* (YLJCC71) failed to improve the production of triterpenes, while overexpression of *ACL2* (YLJCC72) increased the titers of total triterpenoids and betulinic acid 1.23- and 1.30-fold, respectively (Fig. 5). On the other hand, overexpression of all the genes in the β-oxidation pathway except *PEX10* in YLJCC5 led to increased triterpene production. Among the generated strains, YLJCC76 (overexpressing *POT1*) exhibited the most significant enhancement of production with a triterpene yield of 66.77 ± 2.79 mg/L. Similarly, this elevated production did not occur in the strains generated from YLJCC6, which might be caused by module incompatibility.

In most cases, studies have focused on elimination of the competition pathway to reduce carbon loss. For example, α-santalene production increased upon knocking out of citrate synthase or malate synthase, which convert acetyl-CoA to citrate or C4 organic acids, respectively [64]. Fatty acid biosynthesis is also considered to be a competitive pathway for terpenoid synthesis because these pathways use the same precursor—acetyl-CoA. To reduce competition, the endogenous promoters of the key genes in the fatty acid pathway were replaced with relatively weak promoters, and the betulinic acid yield increased to 1.92 mg/L/OD in *S. cerevisiae* [23]. In this study, we applied a different strategy to address competition by enhancing fatty acid β-oxidation to recover acetyl-CoA. The results indicated that the strategy was effective for betulinic acid-related triterpenoid production in *Y. lipolytica*.

### Further increasing the production of triterpenoids by multimodular optimization

**Fig. 6:**
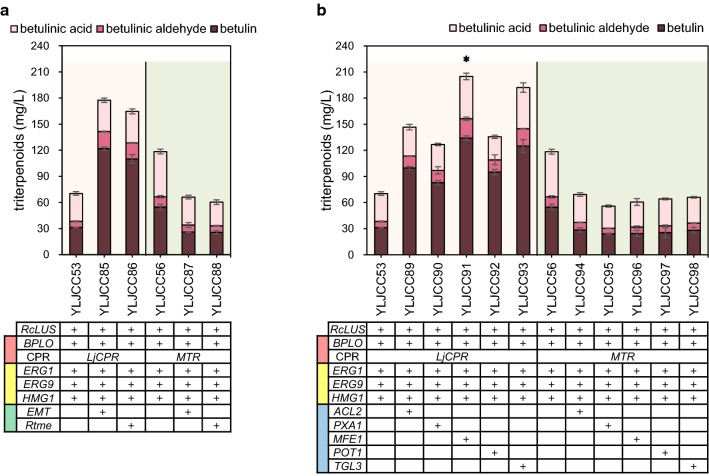
Multimodular optimization to further improve triterpenoid production. **a** Triterpenoid production by strains in which the CYP/CRP, MVA and redox cofactor supply modules were optimized in combination. **b** Triterpenoid production by strains in which the CYP/CPR, MVA and acetyl-CoA generation modules were optimized in combination. The light orange background represents strains generated from YLJCC53, and the reseda background represents strains generated from YLJCC56. Red, yellow, green and blue blocks in the first column of the gene forms represent the CYP/CPR, MVA, redox cofactor supply, and acetyl-CoA generation modules, respectively. Asterisk represents the strain exhibiting the highest total triterpenoid production in this study

By partitioning the betulinic acid biosynthetic pathway into four modules and engineering these modules separately, we identified the effective genes within each module. To boost triterpenoid production, we manipulated these modules in combination. Strains YLJCC53 and YLJCC56 (Fig. 3), which possessed the optimized heterogenous CYP/CPR module and MVA module, were used for the engineering process. *EMT* and *Rtme* in the redox cofactor supply module (Fig. 4) or *ACL2*, *PXA1*, *MFE1*, *POT1*, and *TGL3* in the acetyl-CoA generation module (Fig. 5), which showed the capacity to increase triterpenoid production, were further overexpressed, generating strains YLJCC85-YLJCC98. The fermentation results for these strains are shown in Fig. 6. The strains generated from YLJCC53 showed observable improvement in betulin production, with a 1.8- to 2.9-fold increase in total triterpenoid titers compared with YLJCC53. Among these strains, the highest production was observed for strain YLJCC91, which exhibited a total triterpenoid yield of 204.89 ± 11.56 mg/L; the triterpenoids were composed of 65.44% betulin, 23.71% betulinic acid and 10.85% betulinic aldehyde. Although this dramatic increase was not observed in strains generated from YLJCC56, which might be due to the incompatibility of elevated redox or acetyl-CoA supply with the *BPLO/MTR* module, YLJCC56 exhibited the best betulinic acid production. Previously, the highest betulinic acid and triterpenoid yields in yeast in shake flask cultures were 28 mg/L and 147 mg/L, respectively [22]. Here, via systematic engineering, we obtained two high-yield strains. The first strain was YLJCC56 (Fig. 3), which produced 51.87 ± 2.77 mg/L betulinic acid. The second strain was YLJCC91 (Fig. 6b), which produced 204.89 ± 11.56 mg/L total triterpenoids. To the best of our knowledge, both titers are the highest in microbes in shake flask cultures to date. However, the yield of betulinic acid and total triterpenoids in this study still remained far from the theoretical yield, thus, more studies need to be carried out to improve the heterologous production of those valuable compounds.

**Fig. 7:**
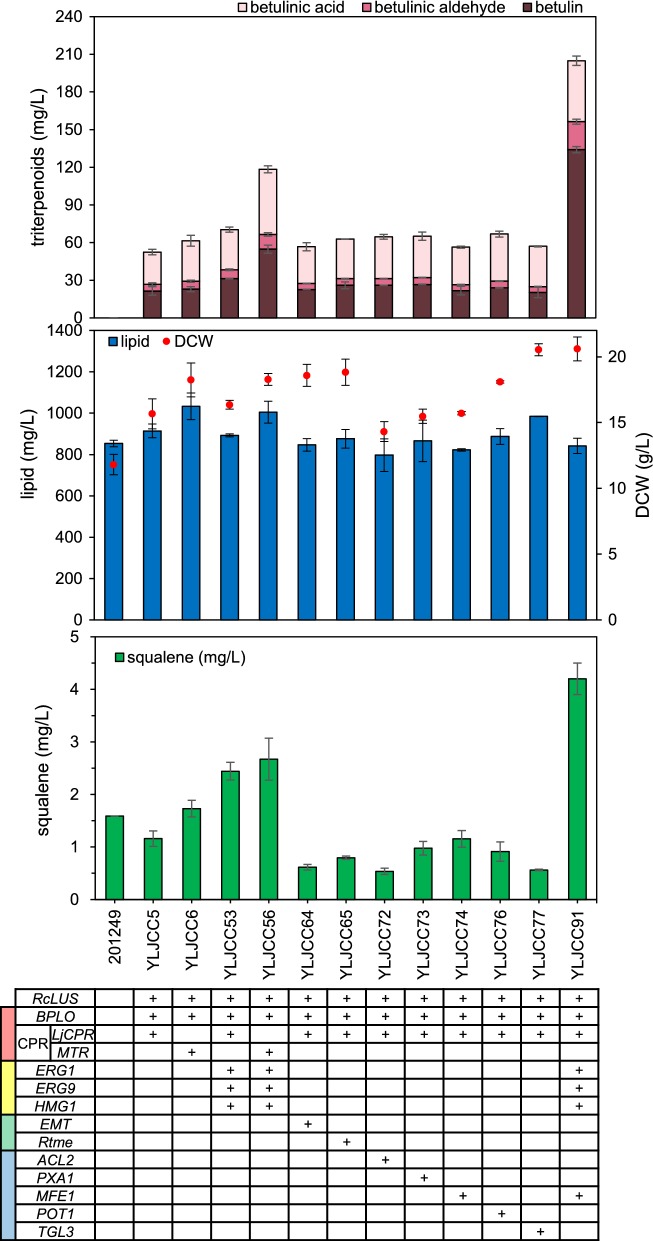
Triterpenoid, lipid, DCW, and squalene production in the parent strain and engineered strains marked with Asterisk. Red, yellow, green and blue blocks in the first column of the gene form represent the CYP/CPR, MVA, redox cofactor supply, and acetyl-CoA generation modules, respectively

To identify the impact of the engineering on cell growth and metabolism, we determined the dry cell weights (DCWs) and the levels of squalene and lipids in the parent strain 201249 and engineered strains marked with * (YLJCC5 and YLJCC6 in Fig. 2a; YLJCC53 and YLJCC56 in Fig. 3; YLJCC64 and YLJCC65 in Fig. 4; YLJCC72, YLJCC73, YLJCC74, YLJCC76, and YLJCC77 in Fig. 5; YLJCC91 in Fig. 6b). The results are presented in Fig. 7. Although the lipid levels and DCWs showed slight differences among the engineered strains, the change pattern was not directly correlated with triterpenoid production (the low biomass of the parent strain 201249 might result from its triple auxotroph characteristics [65]). However, the squalene levels showed a strong correlation with MVA module manipulation. After upregulation of the MVA module, the levels of squalene in YLJCC53 and YLJCC56 increased 1.54- and 1.68-fold, respectively, compared with those in the parent strain 201249. Based on YLJCC53, improvement of acetyl-CoA generation by overexpression of *MFE1* (YLJCC91) further increased squalene accumulation 1.72-fold, which might represent an increased carbon flux to the MVA pathway and terpenoid synthesis.

## Conclusions

Herein, we systematically engineered *Y. lipolytica* using a multimodular strategy to increase triterpenoid production. By matching CYPs and CPRs, both of which originated from five different sources, and coexpressing these proteins with LUS, we established betulinic acid synthesis in *Y. lipolytica*. Then, the MVA module, acetyl-CoA generation module, and redox cofactor supply module were optimized separately. The betulinic acid yield improved to 51.87 ± 2.77 mg/L when *ERG1*, *ERG9*, and *HMG1* in the MVA module were overexpressed with *RcLUS*, *BPLO* and *MTR*. Finally, the optimal modules were integrated, and a 204.89 ± 11.56 mg/L total triterpenoid yield was realized when *RcLUS*, *BPLO* and *LjCPR* were coexpressed with the upregulated MVA (overexpressing *ERG1*, *ERG9* and *HMG1*) and acetyl-CoA generation module (overexpressing *MFE1*). Both betulinic acid and total triterpenoid production achieved the highest reported yields in microbes in shake flask cultures. This study can serve as a foundation for the construction of efficient triterpene production strains of *Y. lipolytica*.

## Methods

### Strains, medium and culture conditions

TransT1 *E. coli* was used for plasmid construction and propagation, and cell growth was performed at 37 °C in Luria–Bertani (LB) broth with 100 mg/L ampicillin or 50 mg/L kanamycin and shaking at 220 rpm. The incipient *Y. lipolytica* strain ATCC 201249 was kindly provided by Professor Ying-Jin Yuan (Tianjin University, China). All the *Y. lipolytica* strains in this article (shown in Table 1) were cultivated at 28 °C with shaking at 250 rpm. Yeast extract-peptone-dextrose (YPD) medium, consisting of 20 g/L glucose, 20 g/L peptone, and 10 g/L yeast extract, was used for transformation, activation and preculture.

**Table 1:** Strains used in this work

Strains	Characteristics	Source
*Y. lipolytica* ATCC 201249	*MATA*, *ura3*-*302, leu2*-*270, lys8*-*11, pex17*-*HA*	Provided by professor Yuan Yingjin (Tianjin University, China)
YLJCC0	201249 Δ*ku70*::*P*_*TEFin*_-*RcLUS*-*acot, hisG*	This work
YLJCC1	YLJCC0 Δ*ku80::P*_*FBAin*_-*BPLO*-*octt, hisG*	This work
YLJCC2	YLJCC1 *IntF::P*_*TEFin*_-*ATR2*-*lip1t*	This work
YLJCC3	YLJCC1 *IntF::P*_*TEFin*_-*CrCPR*-*lip1t*	This work
YLJCC4	YLJCC1 *IntF::P*_*TEFin*_-*GuCPR*-*lip1t*	This work
YLJCC5	YLJCC1 *IntF::P*_*TEFin*_-*LjCPR*-*lip1t*	This work
YLJCC6	YLJCC1 *IntF::P*_*TEFin*_-*MTR*-*lip1t*	This work
YLJCC7	YLJCC0 Δ*ku80::P*_*FBAin*_-*CrAO*-*octt, hisG*	This work
YLJCC8	YLJCC7 *IntF::P*_*TEFin*_-*ATR2*-*lip1t*	This work
YLJCC9	YLJCC7 *IntF::P*_*TEFin*_-*CrCPR*-*lip1t*	This work
YLJCC10	YLJCC7 *IntF::P*_*TEFin*_-*GuCPR*-*lip1t*	This work
YLJCC11	YLJCC7 *IntF::P*_*TEFin*_-*LjCPR*-*lip1t*	This work
YLJCC12	YLJCC7 *IntF::P*_*TEFin*_-*MTR*-*lip1t*	This work
YLJCC13	YLJCC0 Δ*ku80::P*_*FBAin*_-*MtAO12*-*octt, hisG*	This work
YLJCC14	YLJCC13 *IntF::P*_*TEFin*_-*ATR2*-*lip1t*	This work
YLJCC15	YLJCC13 *IntF::P*_*TEFin*_-*CrCPR*-*lip1t*	This work
YLJCC16	YLJCC13 *IntF::P*_*TEFin*_-*GuCPR*-*lip1t*	This work
YLJCC17	YLJCC13 *IntF::P*_*TEFin*_-*LjCPR*-*lip1t*	This work
YLJCC18	YLJCC13 *IntF::P*_*TEFin*_-*MTR*-*lip1t*	This work
YLJCC19	YLJCC0 Δ*ku80::P*_*FBAin*_-*VvAO15*-*octt, hisG*	This work
YLJCC20	YLJCC19 *IntF::P*_*TEFin*_-*ATR2*-*lip1t*	This work
YLJCC21	YLJCC19 *IntF::P*_*TEFin*_-*CrCPR*-*lip1t*	This work
YLJCC22	YLJCC19 *IntF::P*_*TEFin*_-*GuCPR*-*lip1t*	This work
YLJCC23	YLJCC19 *IntF::P*_*TEFin*_-*LjCPR*-*lip1t*	This work
YLJCC24	YLJCC19 *IntF::P*_*TEFin*_-*MTR*-*lip1t*	This work
YLJCC25	YLJCC0 Δ*ku80::P*_*FBAin*_-*VvAO17*-*octt, hisG*	This work
YLJCC26	YLJCC25 *IntF::P*_*TEFin*_-*ATR2*-*lip1t*	This work
YLJCC27	YLJCC25 *IntF::P*_*TEFin*_-*CrCPR*-*lip1t*	This work
YLJCC28	YLJCC25 *IntF::P*_*TEFin*_-*GuCPR*-*lip1t*	This work
YLJCC29	YLJCC25 *IntF::P*_*TEFin*_-*LjCPR*-*lip1t*	This work
YLJCC30	YLJCC25 *IntF::P*_*TEFin*_-*MTR*-*lip1t*	This work
YLJCC31	YLJCC0 Δ*ku80::P*_*FBAin*_-*BPLO*-*LjCPR*-*octt*, *hisG*-*URA3*-*hisG*	This work
YLJCC32	YLJCC0 Δ*ku80::P*_*FBAin*_-*BPLO*-*L1*-*LjCPR*-*octt*, *hisG*-*URA3*-*hisG*	This work
YLJCC33	YLJCC0 Δ*ku80::P*_*FBAin*_-*BPLO*-*L2*-*LjCPR*-*octt*, *hisG*-*URA3*-*hisG*	This work
YLJCC34	YLJCC0 Δ*ku80::P*_*FBAin*_-*BPLO*-*L3*-*LjCPR*-*octt*, *hisG*-*URA3*-*hisG*	This work
YLJCC35	YLJCC0 Δ*ku80::P*_*FBAin*_-*BPLO*-*L4*-*LjCPR*-*octt*, *hisG*-*URA3*-*hisG*	This work
YLJCC36	YLJCC0 Δ*ku80::P*_*FBAin*_-*LjCPR*-*BPLO*-*octt*, *hisG*-*URA3*-*hisG*	This work
YLJCC37	YLJCC0 Δ*ku80::P*_*FBAin*_-*LjCPR*-*L1*-*BPLO*-*octt*, *hisG*-*URA3*-*hisG*	This work
YLJCC38	YLJCC0 Δ*ku80::P*_*FBAin*_-*LjCPR*-*L2*-*BPLO*-*octt*, *hisG*-*URA3*-*hisG*	This work
YLJCC39	YLJCC0 Δ*ku80::P*_*FBAin*_-*LjCPR*-*L3*-*BPLO*-*octt*, *hisG*-*URA3*-*hisG*	This work
YLJCC41	YLJCC0 Δ*ku80::P*_*FBAin*_-*BPLO*-*MTR*-*octt*, *hisG*-*URA3*-*hisG*	This work
YLJCC42	YLJCC0 Δ*ku80::P*_*FBAin*_-*BPLO*-*L1*-*MTR*-*octt*, *hisG*-*URA3*-*hisG*	This work
YLJCC43	YLJCC0 Δ*ku80::P*_*FBAin*_-*BPLO*-*L2*-*MTR*-*octt*, *hisG*-*URA3*-*hisG*	This work
YLJCC44	YLJCC0 Δ*ku80::P*_*FBAin*_-*BPLO*-*L3*-*MTR*-*octt*, *hisG*-*URA3*-*hisG*	This work
YLJCC45	YLJCC0 Δ*ku80::P*_*FBAin*_-*BPLO*-*L4*-*MTR*-*octt*, *hisG*-*URA3*-*hisG*	This work
YLJCC46	YLJCC0 Δ*ku80::P*_*FBAin*_-*MTR*-*BPLO*-*octt*, *hisG*-*URA3*-*hisG*	This work
YLJCC48	YLJCC0 Δ*ku80::P*_*FBAin*_-*MTR*-*L2*-*BPLO*-*octt*, *hisG*-*URA3*-*hisG*	This work
YLJCC49	YLJCC0 Δ*ku80::P*_*FBAin*_-*MTR*-*L3*-*BPLO*-*octt*, *hisG*-*URA3*-*hisG*	This work
YLJCC50	YLJCC0 Δ*ku80::P*_*FBAin*_-*MTR*-*L4*-*BPLO*-*octt*, *hisG*-*URA3*-*hisG*	This work
YLJCC51	YLJCC5 *rDNA::P*_*EXP*_-*ERG1*-*xpr2t*	This work
YLJCC52	YLJCC5 *rDNA::P*_*EXP*_-*ERG1*-*xpr2t*, *P*_*TEFin*_-*ERG9*-*lip2t*	This work
YLJCC53	YLJCC5 *rDNA::P*_*EXP*_-*ERG1*-*xpr2t*, *P*_*TEFin*_-*ERG9*-*lip2t*, *P*_*TEFin*_-*HMG1*-*octt*	This work
YLJCC54	YLJCC6 *rDNA::P*_*EXP*_-*ERG1*-*xpr2t*	This work
YLJCC55	YLJCC6 *rDNA::P*_*EXP*_-*ERG1*-*xpr2t*, *P*_*TEFin*_-*ERG9*-*lip2t*	This work
YLJCC56	YLJCC6 *rDNA::P*_*EXP*_-*ERG1*-*xpr2t*, *P*_*TEFin*_-*ERG9*-*lip2t*, *P*_*TEFin*_-*HMG1*-*octt*	This work
YLJCC63	YLJCC5 *IntE::P*_*TEFin*_-*EMC*-*pex20t*, *hisG*-*URA3*-*hisG*	This work
YLJCC64	YLJCC5 *IntE::P*_*TEFin*_-*EMT*-*pex20t*, *hisG*-*URA3*-*hisG*	This work
YLJCC65	YLJCC5 *IntE::P*_*TEFin*_-*Rtme*-*pex20t*, *hisG*-*URA3*-*hisG*	This work
YLJCC66	YLJCC5 *IntE::P*_*TEFin*_-*Gapc*-*pex20t*, *hisG*-*URA3*-*hisG*	This work
YLJCC67	YLJCC6 *IntE::P*_*TEFin*_-*EMC*-*pex20t*, *hisG*-*URA3*-*hisG*	This work
YLJCC68	YLJCC6 *IntE::P*_*TEFin*_-*EMT*-*pex20t*, *hisG*-*URA3*-*hisG*	This work
YLJCC69	YLJCC6 *IntE::P*_*TEFin*_-*Rtme*-*pex20t*, *hisG*-*URA3*-*hisG*	This work
YLJCC70	YLJCC6 *IntE::P*_*TEFin*_-*Gapc*-*pex20t*, *hisG*-*URA3*-*hisG*	This work
YLJCC71	YLJCC5 *IntE::P*_*TEFin*_-*ACL1*-*pex20t*, *hisG*-*URA3*-*hisG*	This work
YLJCC72	YLJCC5 *IntE::P*_*TEFin*_-*ACL2*-*pex20t*, *hisG*-*URA3*-*hisG*	This work
YLJCC73	YLJCC5 *IntE::P*_*TEFin*_-*PXA1*-*pex20t*, *hisG*-*URA3*-*hisG*	This work
YLJCC74	YLJCC5 *IntE::P*_*TEFin*_-*MFE1*-*pex20t*, *hisG*-*URA3*-*hisG*	This work
YLJCC75	YLJCC5 *IntE::P*_*TEFin*_-*PEX10*-*pex20t*, *hisG*-*URA3*-*hisG*	This work
YLJCC76	YLJCC5 *IntE::P*_*TEFin*_-*POT1*-*pex20t*, *hisG*-*URA3*-*hisG*	This work
YLJCC77	YLJCC5 *IntE::P*_*TEFin*_-*TGL3*-*pex20t*, *hisG*-*URA3*-*hisG*	This work
YLJCC78	YLJCC6 *IntE::P*_*TEFin*_-*ACL1*-*pex20t*, *hisG*-*URA3*-*hisG*	This work
YLJCC79	YLJCC6 *IntE::P*_*TEFin*_-*ACL2*-*pex20t*, *hisG*-*URA3*-*hisG*	This work
YLJCC80	YLJCC6 *IntE::P*_*TEFin*_-*PXA1*-*pex20t*, *hisG*-*URA3*-*hisG*	This work
YLJCC81	YLJCC6 *IntE::P*_*TEFin*_-*MFE1*-*pex20t*, *hisG*-*URA3*-*hisG*	This work
YLJCC82	YLJCC6 *IntE::P*_*TEFin*_-*PEX10*-*pex20t*, *hisG*-*URA3*-*hisG*	This work
YLJCC83	YLJCC6 *IntE::P*_*TEFin*_-*POT1*-*pex20t*, *hisG*-*URA3*-*hisG*	This work
YLJCC84	YLJCC6 *IntE::P*_*TEFin*_-*TGL3*-*pex20t*, *hisG*-*URA3*-*hisG*	This work
YLJCC85	YLJCC53 *IntE::P*_*TEFin*_-*EMT*-*pex20t*, *hisG*-*URA3*-*hisG*	This work
YLJCC86	YLJCC53 *IntE::P*_*TEFin*_-*Rtme*-*pex20t*, *hisG*-*URA3*-*hisG*	This work
YLJCC87	YLJCC56 *IntE::P*_*TEFin*_-*EMT*-*pex20t*, *hisG*-*URA3*-*hisG*	This work
YLJCC88	YLJCC56 *IntE::P*_*TEFin*_-*Rtme*-*pex20t*, *hisG*-*URA3*-*hisG*	This work
YLJCC89	YLJCC53 *IntE::P*_*TEFin*_-*ACL2*-*pex20t*, *hisG*-*URA3*-*hisG*	This work
YLJCC90	YLJCC53 *IntE::P*_*TEFin*_-*PXA1*-*pex20t*, *hisG*-*URA3*-*hisG*	This work
YLJCC91	YLJCC53 *IntE::P*_*TEFin*_-*MFE1*-*pex20t*, *hisG*-*URA3*-*hisG*	This work
YLJCC92	YLJCC53 *IntE::P*_*TEFin*_-*POT1*-*pex20t*, *hisG*-*URA3*-*hisG*	This work
YLJCC93	YLJCC53 *IntE::P*_*TEFin*_-*TGL3*-*pex20t*, *hisG*-*URA3*-*hisG*	This work
YLJCC94	YLJCC56 *IntE::P*_*TEFin*_-*ACL2*-*pex20t*, *hisG*-*URA3*-*hisG*	This work
YLJCC95	YLJCC56 *IntE::P*_*TEFin*_-*PXA1*-*pex20t*, *hisG*-*URA3*-*hisG*	This work
YLJCC96	YLJCC56 *IntE::P*_*TEFin*_-*MFE1*-*pex20t*, *hisG*-*URA3*-*hisG*	This work
YLJCC97	YLJCC56 *IntE::P*_*TEFin*_-*POT1*-*pex20t*, *hisG*-*URA3*-*hisG*	This work
YLJCC98	YLJCC56 *IntE::P*_*TEFin*_-*TGL3*-*pex20t*, *hisG*-*URA3*-*hisG*	This work
YLJCC99	YLJCC0 *IntF::P*_*TEFin*_-*LjCPR*-*lip1t*	This work
YLJCC100	YLJCC0 *IntF::P*_*TEFin*_-*MTR*-*lip1t*	This work
YLJCC101	YLJCC0 Δ*ku80::P*_*FBAin*_-*BPLO*-*ER*-*octt, hisG*; *IntE::P*_*TEFin*_-*LjCPR*-*ER*-*pex20t, hisG*-*URA3*-*hisG*	This work
YLJCC102	YLJCC0 Δ*ku80::P*_*FBAin*_-*BPLO*-*ER*-*octt, hisG*; *IntE::P*_*TEFin*_-*MTR*-*ER*-*pex20t, hisG*-*URA3*-*hisG*	This work
YLJCC103	201249 Δ*ku80::P*_*FBAin*_-*BPLO*-*sfGFP*-*octt, hisG*-*URA3*-*hisG*	This work
YLJCC104	201249 Δ*ku80::P*_*FBAin*_-*LjCPR*-*sfGFP*-*octt, hisG*-*URA3*-*hisG*	This work
YLJCC105	201249 Δ*ku80::P*_*FBAin*_-*MTR*-*sfGFP*-*octt, hisG*-*URA3*-*hisG*	This work

The proper auxotrophic medium used to grow transformants consisted of 20 g/L glucose H_2_O, 5 g/L (NH_4_)_2_SO_4_, 1.7 g/L yeast nitrogen base without amino acids, 2 g/L complete supplement mixture (CSM) lacking uracil (SC-Ura) or leucine (SC-Leu). YPD-hum medium containing 200 mg/L hygromycin was also used to screen the recombined strains, and 20 g/L agar was added for preparation of solid plates. Rich YPD medium, consisting of 50 g/L glucose, 20 g/L peptone, and 10 g/L yeast extract, was used for fermentation.

The seed cultures were first prepared in 25-mL polypropylene tubes containing 5 mL of YPD medium. After 24 h, the seed cultures were inoculated into a 250-mL shake flask containing 50 mL of rich YPD medium with an initial OD_600_ of 0.2 for 168 h. A 1/5 volumetric ratio of isopropyl myristate (IPM) was added to the shake flasks at 120 h to extract the products. All the titers presented in this research were based on the volume of the fermentation media. All assays were performed in at least triplicate (at least three independent assays for three different clones).

### Plasmids and strain construction

All the plasmids used in this study are listed in Table 2. The integration plasmids pIntK, pIntF, pIntE, pK8FB, pLD01, pLD02in, pLD03in, prDNA-hph, pt1-R, pt2-R, and pt3-R were kindly provided by Professor Ying-Jin Yuan (Tianjin University, China). Five CYP-coding genes (*BPLO* from *Betula platyphylla*, *CrAO* from *Catharanthus roseus*, *MtAO12* from *Medicago truncatula*, *VvAO15* and *VvAO17* from *Vitis vinifera*), five CPR-coding genes (*ATR2* from *Arabidopsis thaliana*, *CrCPR* from C*atharanthus roseus*, *GuCPR* from *Glycyrrhiza uralensis*, *LjCPR* from *Lotus japonicas* and *MTR* from *Medicago truncatula*), four genes coding enzymes related to NADH or NADPH generation (*EMC* from *Mucor circinelloides*, *EMT* from *Mortierella alpina*, *Gapc* from *Kluyveromyces lactis* and *Rtme* from *Rhodotorula toruloides NP11*) and *sfGFP* were codon optimized and synthesized by GenScript (Nanjing, China) and listed in Additional file 7: Table S3. Three structural genes of MVA pathway (*ERG1*, *ERG9* and *HMG1*), the long-chain fatty acid transporter coded gene *PXA1*, 3-ketoacyl-CoA thiolase coded gene *POT1*, multifunctional beta-oxidation enzyme coded gene *MFE1*, transcription factor coded gene *PEX10*, ATP citrate lyase subunit a coded gene *ACL1*, ATP citrate lyase subunit b coded gene *ACL2*, and triacyl -glycerol lipase coded gene *TGL3* were amplified from genomic DNA of *Y. lipolytica.* All the genes were amplified with the proper sticky ends and incorporated into the corresponding integration plasmids by Golden Gate assembly or Gibson assembly.

**Table 2:** Plasmids used in this work

Name	Relative characteristics	Source
*Plasmids*		
pIntK	Ku70up-*P*_*TEFin*_-*acot*-hisG-URA3-hisG-Ku70dn cassette in PUC57-1.8k	This work
pK8FB	Ku80up-*P*_*FBAin*_-*octt*-hisG-URA3-hisG-Ku80dn cassette in PUC57-kan	This work
pIntF	IntFup-*P*_*TEFin*_-*lip1t*-LUE-IntFdn cassette in PUC57-1.8k	This work
pIntE	IntEup-*P*_*TEFin*_-*pex20t*-hisG-URA3-hisG-IntEdn cassette in PUC57-1.8k	This work
prDNA-hph	rDNAup-hph	This work
pt1-R	*xpr2t*-rDNAdn	This work
pt2-R	*lip2t*-rDNAdn	This work
pt3-R	*octt*-rDNAdn	This work
pLD01	*P*_*EXP1*_-*xpr2t*	This work
pLD02in	*P*_*TEFin*_-*lip2t*	This work
pLD03in	*P*_*TEFin*_-*octt*	This work
pIntK-RcLUS	*RcLUS* was cloned into pIntK	This work
pK8FB-BPLO	*BPLO* was cloned into pK8FB	This work
pK8FB-CrAO	*CrAO* was cloned into pK8FB	This work
pK8FB-MtAO12	*MtAO12* was cloned into pK8FB	This work
pK8FB-VvAO15	*VvAO15* was cloned into pK8FB	This work
pK8FB-VvAO17	*VvAO17* was cloned into pK8FB	This work
pIntF-ATR2	*ATR2* was cloned into pIntF	This work
pIntF-CrCPR	*CrCPR* was cloned into pIntF	This work
pIntF-GuCPR	*GuCPR* was cloned into pIntF	This work
pIntF-LjCPR	*LjCPR* was cloned into pIntF	This work
pIntF-MTR	*MTR* was cloned into pIntF	This work
pK8FB-BPLO-LjCPR	*BPLO*-*L0*-*LjCPR* was cloned into pK8FB	This work
pK8FB-BPLO-L1-LjCPR	*BPLO*-*L1*-*LjCPR* was cloned into pK8FB	This work
pK8FB-BPLO-L2-LjCPR	*BPLO*-*L2*-*LjCPR* was cloned into pK8FB	This work
pK8FB-BPLO-L3-LjCPR	*BPLO*-*L3*-*LjCPR* was cloned into pK8FB	This work
pK8FB-BPLO-L4-LjCPR	*BPLO*-*L4*-*LjCPR* was cloned into pK8FB	This work
pK8FB-LjCPR-BPLO	*LjCPR*-*L0*-*BPLO* was cloned into pK8FB	This work
pK8FB-LjCPR-L1-BPLO	*LjCPR*-*L1*-*BPLO* was cloned into pK8FB	This work
pK8FB-LjCPR-L2-BPLO	*LjCPR*-*L2*-*BPLO* was cloned into pK8FB	This work
pK8FB-LjCPR-L3-BPLO	*LjCPR*-*L3*-*BPLO* was cloned into pK8FB	This work
pK8FB-BPLO-MTR	*BPLO*-*L0*-*MTR* in pK8FB	This work
pK8FB-BPLO-L1-MTR	*BPLO*-*L1*-*MTR* was cloned into pK8FB	This work
pK8FB-BPLO-L2-MTR	*BPLO*-*L2*-*MTR* was cloned into pK8FB	This work
pK8FB-BPLO-L3-MTR	*BPLO*-*L3*-*MTR* was cloned into pK8FB	This work
pK8FB-BPLO-L4-MTR	*BPLO*-*L4*-*MTR* was cloned into pK8FB	This work
pK8FB-MTR-BPLO	*MTR*-*L0*-*BPLO* was cloned into pK8FB	This work
pK8FB-MTR-L2-BPLO	*MTR*-*L2*-*BPLO* was cloned into pK8FB	This work
pK8FB-MTR-L3-BPLO	*MTR*-*L3*-*BPLO* was cloned into pK8FB	This work
pK8FB-MTR-L4-BPLO	*MTR*-*L4*-*BPLO* was cloned into pK8FB	This work
pLD01-ERG1	*ERG1* was cloned into pLD01	This work
pLD02in-ERG9	*ERG9* was cloned into pLD02in	This work
pLD03in-HMG1	*HMG1* was cloned into pLD03in	This work
pIntE-ACL1	*ACL1* was cloned into pIntE	This work
pIntE-ACL2	*ACL2* was cloned into pIntE	This work
pIntE-PXA1	*PXA1* was cloned into pIntE	This work
pIntE-POT1	*POT1* was cloned into pIntE	This work
pIntE-MFE1	*MFE1* was cloned into pIntE	This work
pIntE-TGL3	*TGL3* was cloned into pIntE	This work
pIntE-PEX10	*PEX10* was cloned into pIntE	This work
pIntE-EMC	*EMC* was cloned into pIntE	This work
pIntE-EMT	*EMT* was cloned into pIntE	This work
pIntE-Gapc	*Gapc* was cloned into pIntE	This work
pIntE-Rtme	*Rtme* was cloned into pIntE	This work
pK8FB-BPLO-ER	*BPLO*-*ER* was cloned into pK8FB	This work
pIntE-LjCPR-ER	*LjCPR*-*ER* was cloned into pIntE	This work
pIntE-MTR-ER	*MTR*-*ER* was cloned into pIntE	This work
pK8FB-BPLO-sfGFP	*BPLO*-*sfGFP* was cloned into pK8FB	This work
pK8FB-LjCPR-sfGFP	*LjCPR*-*sfGFP* was cloned into pK8FB	This work
pK8FB-MTR-sfGFP	*MTR*-*sfGFP* was cloned into pK8FB	This work

Fusion protein-encoding genes were obtained by overlap extension polymerase chain reaction (PCR). All the primers used to construct fusion protein-encoding genes were synthesized by Genewiz Inc. (China) and are listed in Additional file 7: Table S2. We applied two types of fusion between CYP and CPR. In the first case, CYPs were fused with CPRs directly (marked as L0) and generated pK8FB-BPLO-LjCPR, pK8FB-BPLO-MTR, pK8FB-LjCPR-BPLO, and pK8FB-MTR-BPLO. In the other case, CYPs and CPRs were fused with different linkers, which contained linker1 (L1) “GGAGGAGGATCT”, linker2 (L2) “GGATCTGGA, linker3 (L3) “GGAGGAGGAGGATCT”, and linker4 (L4) “GAGGC CGCCGCCAAG” (the first three represents flexible linkers and the last one represents rigid linker), and generated pK8FB-BPLO-L1-LjCPR, pK8FB-BPLO-L2-LjCPR, pK8FB-BPLO-L3-LjCPR, pK8FB-BPLO-L4-LjCPR, pK8FB-LjCPR-L1-BPLO, pK8FB-LjCPR-L2-BPLO, pK8FB-LjCPR-L3-BPLO, pK8FB-BPLO-L1-MTR, pK8FB-BPLO-L2-MTR, pK8FB-BPLO-L3-MTR, pK8FB-BPLO-L4-MTR, pK8FB-MTR-L2-BPLO, pK8FB-MTR-L3-BPLO and pK8FB-MTR-L4-BPLO. All the fragments were amplified with the proper sticky ends and incorporated into the integration plasmid K8FB by Golden Gate assembly. The ER-targeting peptide (KDEL) and sfGFP were fused to the C-terminus of CYP and CPR to generate pK8FB-BPLO-ER, pIntE-LjCPR-ER, pIntE-MTR-ER, and pK8FB-BPLO-sfGFP, pK8FB-LjCPR-sfGFP, and pK8FB-MTR-sfGFP.

All the integrative fragments were transformed into *Y. lipolytica* using the Zymogen Frozen EZ Yeast Transformation Kit II (Zymo Research Corporation). Two micrograms of linearized DNA were used in the transformation reaction, and then, the cells were harvested and plated on selective solid plates. The Ura3 selection maker was recycled by incubating the transformants in 4 mL of YPD for 4 days and diluting 10,000 times on SC solid medium containing 1.2 mg/mL 5-fluoroorotic acid for 2 days. The genotypes of all the strains were confirmed by colony PCR using KOD FX DNA polymerase (Toyobo Co., Ltd.; Shanghai, China). The restriction endonucleases and ligase used for plasmid construction were purchased from NEB. Plasmid extraction and DNA fragment purification were performed using the TIANprep Mini Plasmid Kit and TIANgal Midi Purification Kit, respectively (TIANGEN BIOTECH, China). Gibson assembly was carried out using the ClonExpressII One Step Cloning Kit (Vazyme, China).

### Lupeol, betulinic acid, betulin, and betulinic aldehyde extraction and analysis

The IPM phase was collected by centrifugation of 10 mL of fermentation broth mixture (10 min, 5000 rpm) followed by filtration with a 0.22-μm film. Then, 50 μL of the resulting extract was trimethylsilylated by mixing with 150 μL of *N*-methyl-*N*-(trimethylsilyl) trifluoracetamide (MSTFA) at 80 °C for 1 h and filtering with a 0.22-μm film. Appropriate concentrations of lupeol, betulin (Aladdin Industrial Corporation, US) and betulinic acid (MedChemExpress, US) were dissolved in IPM to prepare standard curves via GC–MS analysis. The methods used for betulinic acid detection were based on previous studies with some modification [15–17, 20]. A Rts-5 MS (30 m × 0.25 mm × 0.5 μm) GC column was used on a SHIMADZU GCMS-QP2020 system. A 1-μL sample was injected by AOC-20i+s with a split ratio of 10. The temperature of the injector was set at 250 °C. The initial oven temperature was maintained at 80 °C for 2 min, and then, the temperature was increased to 300 °C at a rate of 20 °C/min and held for 17 min. The GC system was operated under a constant pressure of 91 kPa. For the MS system, the temperature of the ion source was 230 °C, and full scan mode was used from 50 to 700 m/z.

### Squalene extraction and analysis

Extraction and analysis of squalene were performed according to Guo XJ et al. [66] with some modification. Cells were harvested by centrifugation and washed 3 times with distilled water. Then, 1 mL of 3 M HCl was added for resuspension, followed by boiling in a 100 °C water bath for 5 min. The cell debris was washed with distilled water until the pH was neutral. NaOH solution was used to neutralize the residual HCl. Then, the cell pellet was resuspended with 1.5 M NaOH-methanol solution and incubated at 60 °C for 4 h. Then, 500 μL of hexyl hydride was added to the tubes, and the mixture was vortexed for 20 min. After centrifugation, the hexyl hydride layer was transferred to new tubes. This process was repeated once. The hexyl hydride layer was vacuum-dried, resuspended in 100 μL of MSTFA and incubated at 30 °C for 2 h. After filtering with a 0.22-μm film, the sample was used for GC–MS detection. A Rts-5 MS (30 m × 0.25 mm × 0.5 μm) GC column was used on the SHIMADZU GCMS-QP2020 system. A 1-μL sample was injected by AOC-20i + s with a split ratio of 5. The temperature of the injector was set at 280 °C. The initial oven temperature was maintained at 70 °C for 1 min, and then, the temperature was increased to 250 °C at a rate of 30 °C/min and held for 2 min. Then, the temperature was increased to 280 °C at a rate of 20 °C/min and held for 20 min. The GC system was operated under a constant pressure of 91 kPa. For the MS system, the temperature of the ion source was 220 °C, and full scan mode was used from 50 to 600 m/z.

### Lipid extraction and analysis

Extraction and analysis of lipids were performed according to Wang et al. [67]. Cells were harvested by centrifugation and washed 3 times with distilled water. One milliliter of methanol containing 3 M HCl and 100 mg/L heptadecanoic acid (as an internal standard) was added to the tubes and vortexed for mixing. Then, these tubes were incubated at 70 °C for 3 h and inverted several times for 40 min. Then, these tubes were cooled naturally to the indoor temperature. Then, sodium chloride was added into the solutions until saturation, and the solutions were shaken for 1 min. Then, 500 μL of hexyl hydride was added into these tubes with shaking for 1 min. The hexyl hydride phase was collected by centrifugation, filtered with a 0.22-μm film and used for the detection of lipid production by GC–MS. A Rts-5 MS (30 m × 0.25 mm × 0.5 μm) GC column was used on a SHIMADZU GCMS-QP2020 system. A 1-μL sample was injected by AOC-20i+s with a split ratio of 50. The temperature of the injector was set at 260 °C. The initial oven temperature was maintained at 70 °C for 2 min, and then, the temperature was increased to 290 °C at a rate of 8 °C/min and held for 6 min. The GC system was operated under a constant pressure of 91 kPa. For the MS system, the temperature of the ion source was 220 °C, and full scan mode was used from 50 to 500 m/z.

### Phylogenetic tree and homology analyses

Full-length amino acid sequences were obtained from GenBank and aligned by ClustalW. A phylogenetic tree was constructed using the neighbor-joining method with 1000 replicates using MEGA6 software. Amino acid sequence homology analysis was performed with BLASTP.

### Confocal laser scanning microscopy

Cell imaging was performed using UltraView Vox (PerkingElmer, America) after washing with ddH2O. Excitation/emission wavelengths were 488 nm/530 nm for GFP.

## Additional files

**Additional file 1: Figure S1.** Structures of the endogenous compound 2,3-oxidosqualene and the heterogenous compounds lupeol, betulin, betulinic aldehyde and betulinic acid.

**Additional file 2: Figure S2.** Production of lupeol in the parent strain 201249 and YLJCC0 (expressing *RcLUS* from *R. communis*).

**Additional file 3: Table S1.** Triterpenoid production by strains with or without introduction of a plant CPR (LiCPR or MTR). No matter whether heterologous CPRs were expressed, no betulinic acid was observed, which indicated that the native P450 enzymes in *Y. lipolytica* did not catalyze betulinic acid production.

**Additional file 4: Figure S3.** GC–MS profiles and mass spectra of chemical standards and fermentation extracts from YLJCC6. (a) GC–MS profiles of the (1) lupeol standard, (2) betulin standard, (3) betulinic acid standard, and (4) metabolite extracts of YLJCC6. (b) Mass spectra of lupeol and betulinic acid. Red represents the mass spectrum of the metabolite extracts of YLJCC6, and gray represents the mass spectrum of the standards.

**Additional file 5: Figure S4.** (a) Phylogenetic tree of plant CYPs that were identified or deduced as lupeol c-28 oxidases constructed by MEGA6. (b) Homology analysis of CrAO, MtAO12, VvAO15 and VvAO17 with BPLO by BLASTP.

**Additional file 6: Figure S5.** Confocal image of strains that expressed CYP or CPR fused with sfGFP. sfGFP was fused to the C-terminus of CYP or CPR.

**Additional file 7: Table S2.** Oligonucleotides used in construct plasmids in this study. **Table S3.** The Codon-optimized gene sequences involved in this study.
