# Supplementary figures and images for: Boosting the biosynthesis of betulinic acid and related triterpenoids in Yarrowia lipolytica via multimodular metabolic engineering
Source: Microb Cell Fact. 2019 May 3;18:77. doi: 10.1186/s12934-019-1127-8 (PMC6498500; doi:10.1186/s12934-019-1127-8)

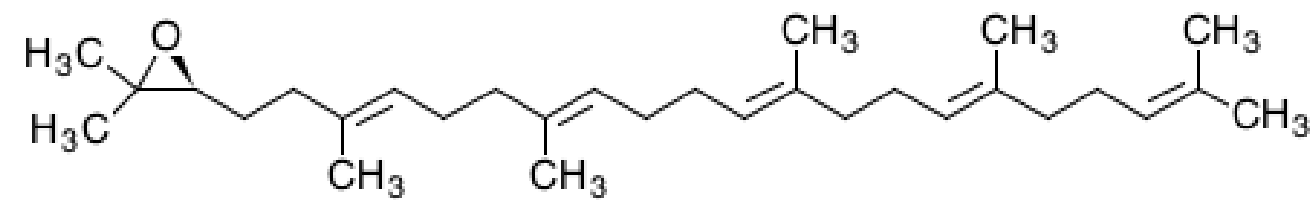

2,3-Oxidosqualene

LUS

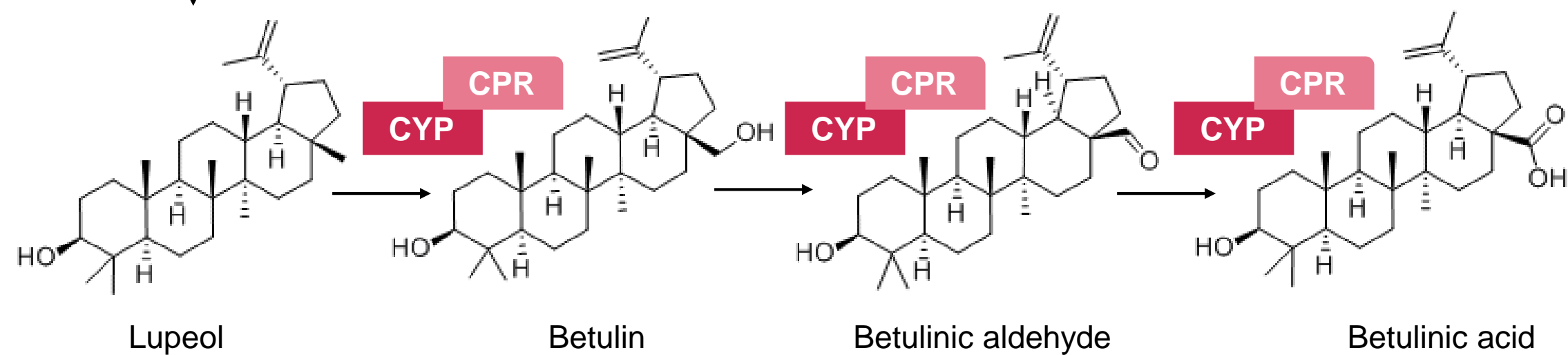

Supplement: Supplementary file 1 — Additional file 1: Figure S1. Structures of the endogenous compound 2,3-oxidosqualene and the heterogenous compounds lupeol, betulin, betulinic aldehyde and betulinic acid. [file 12934_2019_1127_MOESM1_ESM.pdf]

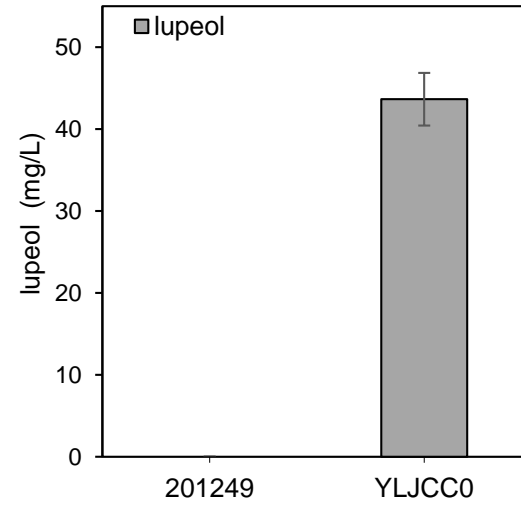

Supplement: Supplementary file 2 — Additional file 2: Figure S2. Production of lupeol in the parent strain 201249 and YLJCC0 (expressing RcLUS from R. communis). [file 12934_2019_1127_MOESM2_ESM.pdf]

(a)

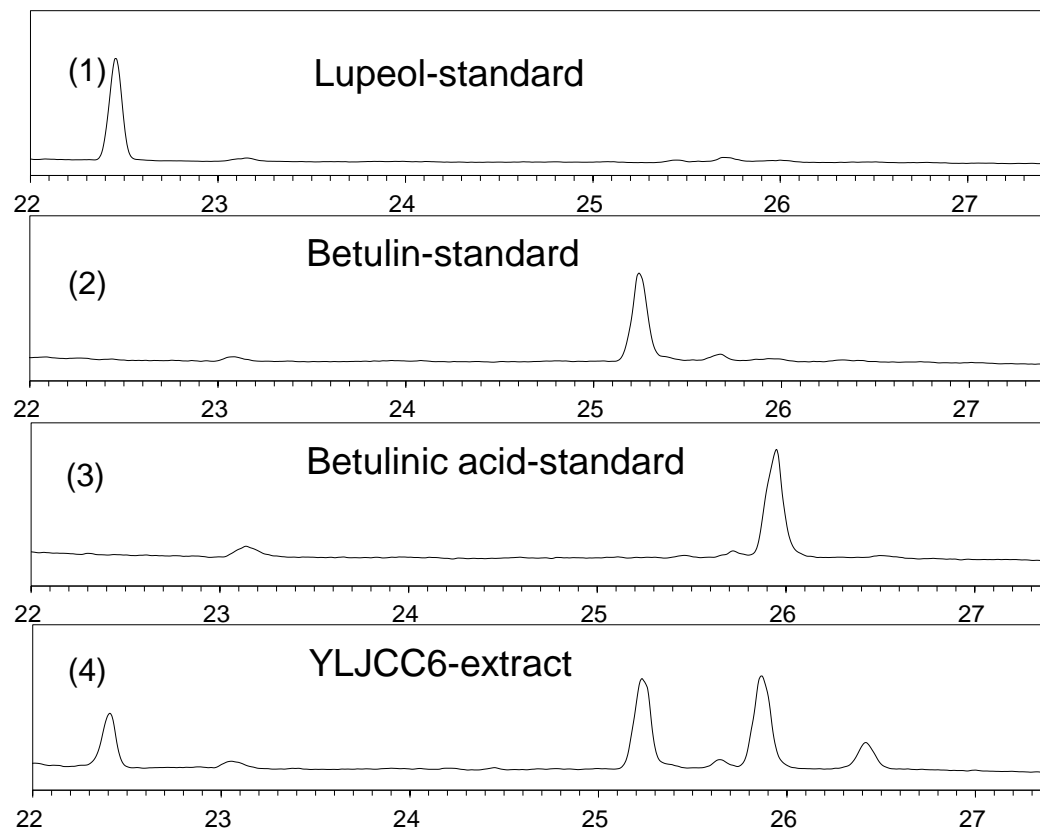

(b)

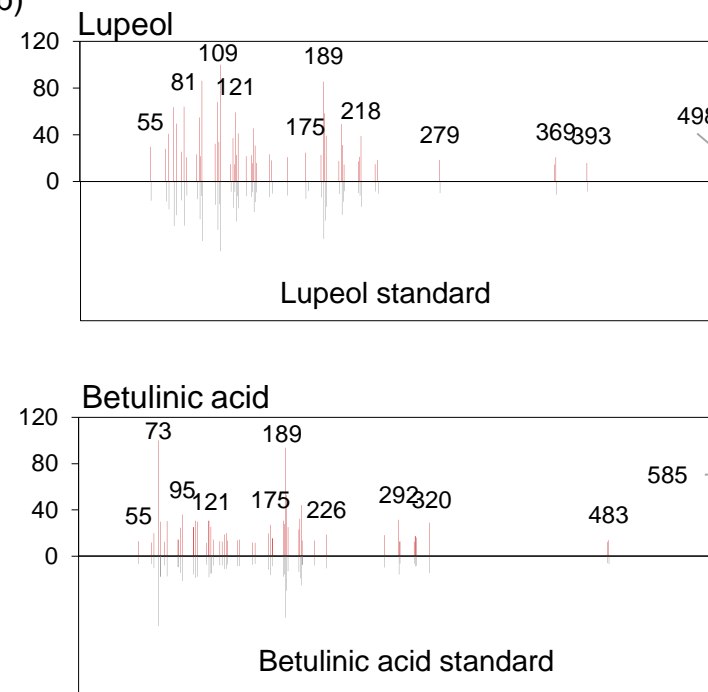

Supplement: Supplementary file 4 — Additional file 4: Figure S3. GC–MS profiles and mass spectra of chemical standards and fermentation extracts from YLJCC6. (a) GC–MS profiles of the (1) lupeol standard, (2) betulin standard, (3) betulinic acid standard, and (4) metabolite extracts of YLJCC6. (b) Mass spectra of lupeol and betulinic acid. Red represents the mass spectrum of the metabolite extracts of YLJCC6, and gray represents the mass spectrum of the standards. [file 12934_2019_1127_MOESM4_ESM.pdf]

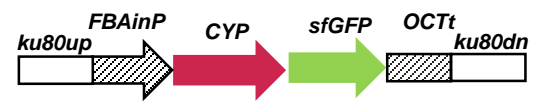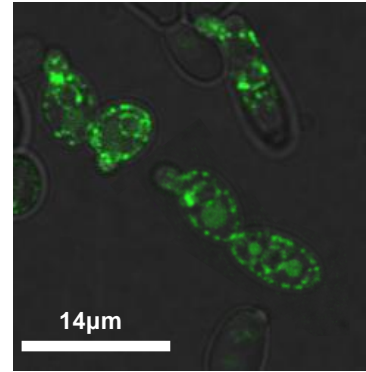

**BPLO-sfGFP**

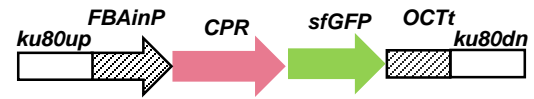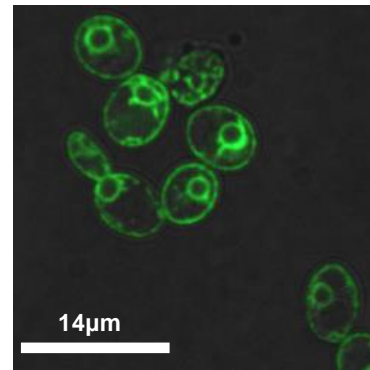

**LjCPR-sfGFP**

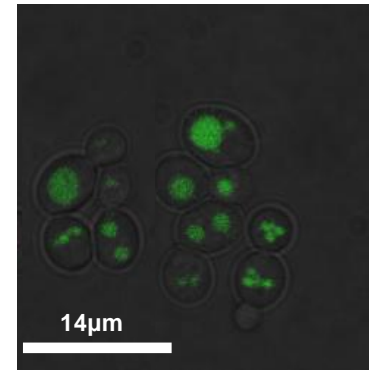

**MTR-sfGFP**

Supplement: Supplementary file 6 — Additional file 6: Figure S5. Confocal image of strains that expressed CYP or CPR fused with sfGFP. sfGFP was fused to the C-terminus of CYP or CPR. [file 12934_2019_1127_MOESM6_ESM.pdf]
