# Supplementary material for: Boosting the biosynthesis of betulinic acid and related triterpenoids in Yarrowia lipolytica via multimodular metabolic engineering
Source: Microb Cell Fact. 2019 May 3;18:77. doi: 10.1186/s12934-019-1127-8 (PMC6498500; doi:10.1186/s12934-019-1127-8)
Supplement: Supplementary file 3 — Additional file 3: Table S1. Triterpenoid production by strains with or without introduction of a plant CPR (LiCPR or MTR). No matter whether heterologous CPRs were expressed, no betulinic acid was observed, which indicated that the native P450 enzymes in Y. lipolytica did not catalyze betulinic acid production. [file 12934_2019_1127_MOESM3_ESM.docx]

**Table S1**

Triterpenoid production by strains with or without introduction of plant CPR (LiCPR or MTR)

| Strain | Heterologous  LUS | Heterologous  CPR | Heterologous CYP | Betulinic acid (mg/L) | Total triterpenoids (mg/L) |
| --- | --- | --- | --- | --- | --- |
| 201249 | - | - | - | 0 | 0 |
| YLJCC0 | RcLUS | - | - | 0 | 0 |
| YLJCC99 | RcLUS | LjCPR | - | 0 | 0 |
| YLJCC100 | RcLUS | MTR | - | 0 | 0 |
| YLJCC5 | RcLUS | LjCPR | BPLO | 25.62 | 54.42 |
| YLJCC6 | RcLUS | MTR | BPLO | 32.33 | 61.49 |
| YLJCC23 | RcLUS | LjCPR | VvAO15 | 12.68 | 41.52 |
| YLJCC24 | RcLUS | MTR | VvAO15 | 17.76 | 50.88 |
