# Supplementary material for: Boosting the biosynthesis of betulinic acid and related triterpenoids in Yarrowia lipolytica via multimodular metabolic engineering
Source: Microb Cell Fact. 2019 May 3;18:77. doi: 10.1186/s12934-019-1127-8 (PMC6498500; doi:10.1186/s12934-019-1127-8)
Supplement: Supplementary file 5 — Additional file 5: Figure S4. (a) Phylogenetic tree of plant CYPs that were identified or deduced as lupeol c-28 oxidases constructed by MEGA6. (b) Homology analysis of CrAO, MtAO12, VvAO15 and VvAO17 with BPLO by BLASTP. [file 12934_2019_1127_MOESM5_ESM.pdf]

(a)

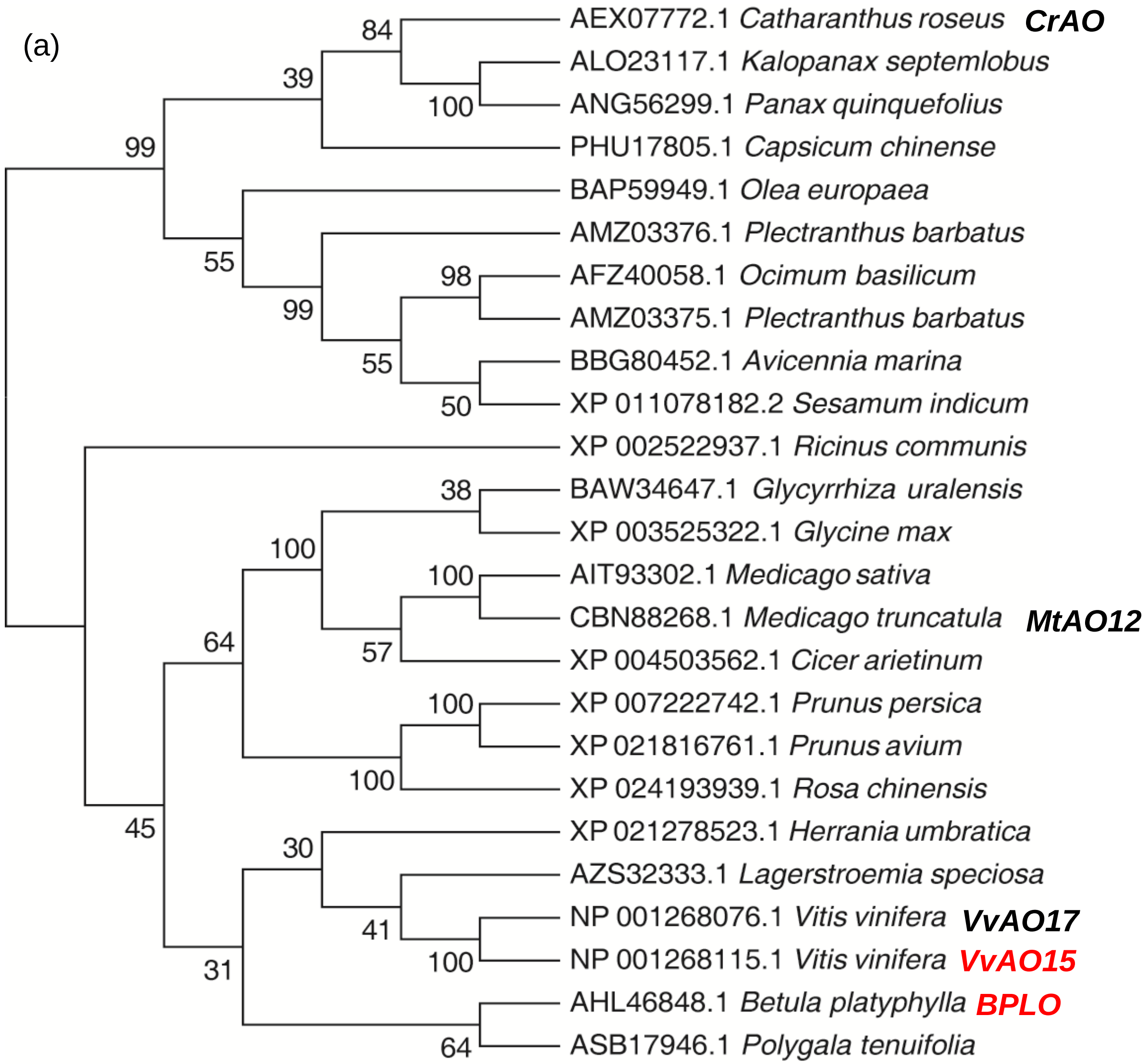


(b)

| Gene | Accession | source | Total score | Identity |
|---|---|---|---|---|
| *BPLO* | AHL46848.1 | *Betula platyphylla* | 997 | 100% |
| *VvAO15* | NP_001268115.1 | *Vitis vinifera* | 833 | 81.04% |
| *VvAO17* | NP_001268076.1 | *Vitis vinifera* | 817 | 81.25% |
| *MtAO12* | CBN88268.1 | *Medicago truncatula* | 796 | 78.50% |
| *CrAO* | AEX07772.1 | *Catharanthus roseus* | 785 | 78.91% |
