# Supplementary material for: Boosting the biosynthesis of betulinic acid and related triterpenoids in Yarrowia lipolytica via multimodular metabolic engineering
Source: Microb Cell Fact. 2019 May 3;18:77. doi: 10.1186/s12934-019-1127-8 (PMC6498500; doi:10.1186/s12934-019-1127-8)
Supplement: Supplementary file 7 — Additional file 7: Table S2. Oligonucleotides used in construct plasmids in this study. Table S3. The Codon-optimized gene sequences involved in this study. [file 12934_2019_1127_MOESM7_ESM.docx]

| **Table S2**  Oligonucleotides used in construct plasmids in this study | |
| --- | --- |
| Oligos | Sequence (5'-3') |
| **Primers for plasmid construction containing gene *RcLUS*** | |
| BsaI-IntK-RcLUS-F | ggtctccgcagtggcgaatcaagatcgccgag |
| BsaI-IntK-RcLUS-R | ggtctcctttacaggttgttggtgaagccg |
| **Primers for plasmid construction containing genes encoding CYPs** | |
| **For construction of pK8FB-BPLO** | |
| BsaI-K8FB-BPLO-F | ggtctccccaggagcacttctacctgtctctgctg |
| BsaI-K8FB-BPLO-R | ggtctcctttaggccttatgagggtacagtcgcac |
| **For construction of pK8FB-CrAO** | |
| BsaI-K8FB-CrAO-F | ggtctccccaggagatcttctacgtgaccctgc |
| BsaI-K8FB-CrAO-R | ggtctcctttaggcattgatgtgggggtaaagtc |
| **For construction of pK8FB-MtAO12** | |
| BsaI-K8FB-MtAO12-F | ggtctccccaggagcccaacttctacctgtctctgc |
| BsaI-K8FB-MtAO12-R | ggtctcctttaggctttgtggggatacagtcgg |
| **For construction of pK8FB-VvAO15** | |
| BsaI-K8FB-VvAO15-F | ggtctccccaggaggtgttcttcctgtctctgctg |
| BsaI-K8FB-VvAO15-R | ggtctcctttaaggtttgtgagggtgcagtctcac |
| **For construction of pK8FB-VvAO17** | |
| BsaI-K8FB-VvAO17-F | ggtctccccaggaggtgttcttcctgtctctgctg |
| BsaI-K8FB-VvAO17-R | ggtctcctttaaggtttgtggggatgcagtctc |
| **Primers for plasmid construction containing genes encoding CPRs** | |
| **For construction of pIntF-ATR2** | |
| BsaI-IntF-ATR2-F | ggtctccgcagaagaacatgatgaactacaagctgaagc |
| BsaI-IntF-ATR2-R | ggtctcctttaccacacatctcgcaggtatcgac |
| **For construction of pIntF-CrCPR** | |
| BsaI-IntF-CrCPR-F | ggtctccgcaggactcttcttctgagaagctgtctcc |
| BsaI-IntF-CrCPR-R | ggtctcctttaccacacatctcgcaggtatctgc |
| **For construction of pIntF-GuCPR** | |
| BsaI-IntF-GuCPR-F | ggtctccgcagacctccaactctgacctggtcc |
| BsaI-IntF-GuCPR-R | ggtctcctttaccacacatctcgcaggtatcgg |
| **For construction of pIntF-LjCPR** | |
| BsaI-IntF-LjCPR-F | ggtctccgcaggaggagtcttcttctatgaagatctctccc |
| BsaI-IntF-LjCPR-R | ggtctcctttaccacacatctcgcaggtatcgg |
| **For construction of pIntF-MTR** | |
| BsaI-IntF-MTR-F | ggtctccgcagacctcttccaactccgacctgg |
| BsaI-IntF-MTR-R | ggtctcctttaccacacatctcgcaggtatcgg |
| **Primers for plasmid construction containing genes in MVA pathway** | |
| **For construction of pLD01-ERG1** | |
| BsaI-LD01-ERG1-F | ggtctccaatggtgactcaacaatctgccgcc |
| BsaI-LD01-ERG1-R | ggtctcctttaggtcagctcagaccagatgtagg |
| **For construction of pLD02in-ERG9** | |
| BsaI-LD02in-ERG9-F | ggtctccgcagggcaagctgattgagctgct |
| BsaI-LD02in-ERG9-R | ggtctcctttagtctcgcagggggaacatc |
| **For construction of pLD03in-HMG1** | |
| BsaI-LD03in-HMG1-F | ggtctccgcagctacaagcagctattggaaagattgtgg |
| BsaI-LD03in-HMG1-R | ggtctcctttatgaccgtatgcaaatattcgaaccgt |
| **Primers for plasmid construction containing genes involved in NADH or NADPH generation** | |
| **For construction of pIntE-EMC** | |
| BsaI-IntE-EMC-F | ggtctccgcagtctcccatcattgacttcgtgc |
| BsaI-IntE-EMC-R | ggtctcctttacagcttgccagcctgctg |
| **For construction of pIntE-EMT** | |
| BsaI-IntE-EMT-F | ggtctccgcaggctctgtcttccctgtccacc |
| BsaI-IntE-EMT-R | ggtctcctttacaggtggggggcgaagg |
| **For construction of pIntE-Rtme** | |
| BsaI-IntE-Rtme-F | ggtctccgcagcctgctcacttcgctccctc |
| BsaI-IntE-Rtme-R | ggtctcctttactgagcctgctgctcgg |
| **For construction of pIntE-Gapc** | |
| BsaI-IntE-Gapc-F | ggtctccgcagcccgatatgaccaacgagtcctc |
| BsaI-IntE-Gapc-R | ggtctcctttacacgccggcctcgaagt |
| **Primers for plasmid construction containing genes encoding fusion proteins of CYP and CPR** | |
| **For construction of pk8FB-BPLO-L0-LjCPR** | |
| BsaI-K8FB-BPLO-F | ggtctccccaggagcacttctacctgtctctgc |
| BPLO-(L0-LjCPR)-R | caggtccaggggagagatcttcatagaagaagactcctcggccttatgagggtacagtcgc |
| LjCPR-F | gaggagtcttcttctatgaagatctctccc |
| BsaI-K8FB-LjCPR-R | ggtctcctttaccacacatctcgcaggtatcgg |
| **For construction of pk8FB-BPLO-L0-MTR** | |
| BsaI-K8FB-BPLO-F | ggtctccccaggagcacttctacctgtctctgc |
| BPLO-(L0-MTR)-R | cagactcgatggttcggaccaggtcggagttggaagaggtggccttatgagggtacagtcgc |
| MTR-F | acctcttccaactccgacctgg |
| BsaI-K8FB-MTR-R | ggtctcctttaccacacatctcgcaggtatcgg |
| **For construction of pk8FB-BPLO-L1-LjCPR** | |
| BsaI-K8FB-BPLO-F | ggtctccccaggagcacttctacctgtctctgc |
| BPLO-(L1-LjCPR)-R | gggagagatcttcatagaagaagactcctcagatcctcctccggccttatgagggtacagtcgc |
| L1-LjCPR-F | ggaggaggatctgaggagtcttcttctatgaagatctctccc |
| BsaI-K8FB-LjCPR-R | ggtctcctttaccacacatctcgcaggtatcgg |
| **For construction of pk8FB-BPLO-L1-MTR** | |
| BsaI-K8FB-BPLO-F | ggtctccccaggagcacttctacctgtctctgc |
| BPLO-(L1-MTR)-R | ccaggtcggagttggaagaggtagatcctcctccggccttatgagggtacagtcgc |
| L1-MTR-F | ggaggaggatctacctcttccaactccgacctgg |
| BsaI-K8FB-MTR-R | ggtctcctttaccacacatctcgcaggtatcgg |
| **For construction of pk8FB-BPLO-L2-LjCPR** | |
| BsaI-K8FB-BPLO-F | ggtctccccaggagcacttctacctgtctctgc |
| BPLO-(L2-LjCPR)-R | gggagagatcttcatagaagaagactcctctccagatccggccttatgagggtacagtcgc |
| L2-LjCPR-F | ggatctggagaggagtcttcttctatgaagatctctccc |
| BsaI-K8FB-LjCPR-R | ggtctcctttaccacacatctcgcaggtatcgg |
| **For construction of pk8FB-BPLO-L2-MTR** | |
| BsaI-K8FB-BPLO-F | ggtctccccaggagcacttctacctgtctctgc |
| BPLO-(L2-MTR)-R | ccaggtcggagttggaagaggttccagatccggccttatgagggtacagtcgc |
| L2-MTR-F | ggatctggaacctcttccaactccgacctgg |
| BsaI-K8FB-MTR-R | ggtctcctttaccacacatctcgcaggtatcgg |
| **For construction of pk8FB-BPLO-L3-LjCPR** | |
| BsaI-K8FB-BPLO-F | ggtctccccaggagcacttctacctgtctctgc |
| BPLO-(L3-LjCPR)-R | gggagagatcttcatagaagaagactcctcagatcctcctcctccggccttatgagggtacagtcgc |
| L3-LjCPR-F | ggaggaggaggatctgaggagtcttcttctatgaagatctctccc |
| BsaI-K8FB-LjCPR-R | ggtctcctttaccacacatctcgcaggtatcgg |
| **For construction of pk8FB-BPLO-L3-MTR** | |
| BsaI-K8FB-BPLO-F | ggtctccccaggagcacttctacctgtctctgc |
| BPLO-(L3-MTR)-R | ccaggtcggagttggaagaggtagatcctcctcctccggccttatgagggtacagtcgc |
| L3-MTR-F | ggaggaggaggatctacctcttccaactccgacctgg |
| BsaI-K8FB-MTR-R | ggtctcctttaccacacatctcgcaggtatcgg |
| **For construction of pk8FB-BPLO-L4-LjCPR** | |
| BsaI-K8FB-BPLO-F | ggtctccccaggagcacttctacctgtctctgc |
| BPLO-(L4-LjCPR)-R | gggagagatcttcatagaagaagactcctccttggcggcggcctcggccttatgagggtacagtcgc |
| L4-LjCPR-F | gaggccgccgccaaggaggagtcttcttctatgaagatctctccc |
| BsaI-K8FB-LjCPR-R | ggtctcctttaccacacatctcgcaggtatcgg |
| **For construction of pk8FB-BPLO-L4-MTR** | |
| BsaI-K8FB-BPLO-F | ggtctccccaggagcacttctacctgtctctgc |
| BPLO-(L4-MTR)-R | ccaggtcggagttggaagaggtcttggcggcggcctcggccttatgagggtacagtcgc |
| L4-MTR-F | gaggccgccgccaagacctcttccaactccgacctgg |
| BsaI-K8FB-MTR-R | ggtctcctttaccacacatctcgcaggtatcgg |
| **For construction of pk8FB-LjCPR-L0-BPLO** | |
| BsaI-K8FB-LjCPR-F | ggtctccccaggaggagtcttcttctatgaagatctctccc |
| LjCPR-(L0-BPLO)-R | gacacgaacagcagcagcagagacaggtagaagtgctcccacacatctcgcaggtatcg |
| BPLO-F | gagcacttctacctgtctctgctg |
| BsaI-K8FB-BPLO-R | ggtctcctttaggccttatgagggtacagtcgcac |
| **For construction of pk8FB-MTR-L0-BPLO** | |
| BsaI-K8FB-MTR-F | ggtctccccagacctcttccaactccgacctgg |
| MTR-(L0-BPLO)-R | gacacgaacagcagcagcagagacaggtagaagtgctcccacacatctcgcaggtatcg |
| BPLO-F | gagcacttctacctgtctctgctg |
| BsaI-K8FB-BPLO-R | ggtctcctttaggccttatgagggtacagtcgcac |
| **For construction of pk8FB-LjCPR-L1-BPLO** | |
| BsaI-K8FB-LjCPR-F | ggtctccccaggaggagtcttcttctatgaagatctctccc |
| LjCPR-(L1-BPLO)-R | cagcagagacaggtagaagtgctcagatcctcctccccacacatctcgcaggtatcg |
| L1-BPLO-F | ggaggaggatctgagcacttctacctgtctctgctg |
| BsaI-K8FB-BPLO-R | ggtctcctttaggccttatgagggtacagtcgcac |
| **For construction of pk8FB-MTR-L1-BPLO** | |
| BsaI-K8FB-MTR-F | ggtctccccagacctcttccaactccgacctgg |
| MTR-(L1-BPLO)-R | cagcagagacaggtagaagtgctcagatcctcctccccacacatctcgcaggtatcg |
| L1-BPLO-F | ggaggaggatctgagcacttctacctgtctctgctg |
| BsaI-K8FB-BPLO-R | ggtctcctttaggccttatgagggtacagtcgcac |
| **For construction of pk8FB-LjCPR-L2-BPLO** | |
| BsaI-K8FB-LjCPR-F | ggtctccccaggaggagtcttcttctatgaagatctctccc |
| LjCPR-(L2-BPLO)-R | cagcagagacaggtagaagtgctctccagatccccacacatctcgcaggtatcg |
| L2-BPLO-F | ggatctggagagcacttctacctgtctctgctg |
| BsaI-K8FB-BPLO-R | ggtctcctttaggccttatgagggtacagtcgcac |
| **For construction of pk8FB-MTR-L2-BPLO** | |
| BsaI-K8FB-MTR-F | ggtctccccagacctcttccaactccgacctgg |
| MTR-(L2-BPLO)-R | cagcagagacaggtagaagtgctctccagatccccacacatctcgcaggtatcg |
| L2-BPLO-F | ggatctggagagcacttctacctgtctctgctg |
| BsaI-K8FB-BPLO-R | ggtctcctttaggccttatgagggtacagtcgcac |
| **For construction of pk8FB-LjCPR-L3-BPLO** | |
| BsaI-K8FB-LjCPR-F | ggtctccccaggaggagtcttcttctatgaagatctctccc |
| LjCPR-(L3-BPLO)-R | cagcagagacaggtagaagtgctcagatcctcctcctccccacacatctcgcaggtatcg |
| L3-BPLO-F | ggaggaggaggatctgagcacttctacctgtctctgctg |
| BsaI-K8FB-BPLO-R | ggtctcctttaggccttatgagggtacagtcgcac |
| **For construction of pk8FB-MTR-L3-BPLO** | |
| BsaI-K8FB-MTR-F | ggtctccccagacctcttccaactccgacctgg |
| MTR-(L3-BPLO)-R | cagcagagacaggtagaagtgctcagatcctcctcctccccacacatctcgcaggtatcg |
| L3-BPLO-F | ggaggaggaggatctgagcacttctacctgtctctgctg |
| BsaI-K8FB-BPLO-R | ggtctcctttaggccttatgagggtacagtcgcac |
| **For construction of pk8FB-LjCPR-L4-BPLO** | |
| BsaI-K8FB-LjCPR-F | ggtctccccaggaggagtcttcttctatgaagatctctccc |
| LjCPR-(L4-BPLO)-R | cagcagagacaggtagaagtgctccttggcggcggcctcccacacatctcgcaggtatcg |
| L4-BPLO-F | gaggccgccgccaaggagcacttctacctgtctctgctg |
| BsaI-K8FB-BPLO-R | ggtctcctttaggccttatgagggtacagtcgcac |
| **For construction of pk8FB-MTR-L4-BPLO** | |
| BsaI-K8FB-MTR-F | ggtctccccagacctcttccaactccgacctgg |
| MTR-(L4-BPLO)-R | cagcagagacaggtagaagtgctccttggcggcggcctcccacacatctcgcaggtatcg |
| L4-BPLO-F | gaggccgccgccaaggagcacttctacctgtctctgctg |
| BsaI-K8FB-BPLO-R | ggtctcctttaggccttatgagggtacagtcgcac |
| **Primers for plasmid construction containing genes encoding endoplasmic reticulum located CYP or CPR** | |
| BsaI-K8FB-BPLO-F | ggtctccccaggagcacttctacctgtctctgc |
| BsaI-ER-BPLO-R | ggtctcctttacagctcgtccttggccttatgagggtacagtcgc |
| BsaI-IntE-LjCPR-F | ggtctccgcaggaggagtcttcttctatgaagatctctccc |
| BsaI-ER-LjCPR-R | ggtctcctttacagctcgtccttccacacatctcgcaggtatcgg |
| BsaI-IntE-MTR-F | ggtctccgcagacctcttccaactccgacctgg |
| BsaI-ER-MTR-R | ggtctcctttacagctcgtccttccacacatctcgcaggtatcgg |
| **Primers for plasmid construction containing genes encoding fusion proteins of CYP and GFP or CPR and GFP** | |
| BsaI-K8FB-BPLO-F | ggtctccccaggagcacttctacctgtctctgctg |
| BPLO-(sfGFP)-R | atgggcaccacaccggtgaacagctcctcaccctttcgggccttatgagggtacagtcgc |
| BsaI-K8FB-LjCPR-F | ggtctccccaggaggagtcttcttctatgaagatctctcc |
| LjCPR-(sfGFP)-R | gatgggcaccacaccggtgaacagctcctcaccctttcgccacacatctcgcaggtatcg |
| BsaI-K8FB-MTR-F | ggtctccccagacctcttccaactccgacctgg |
| MTR-(sfGFP)-R | gatgggcaccacaccggtgaacagctcctcaccctttcgccacacatctcgcaggtatcg |
| sfGFP-F | cgaaagggtgaggagctgttcac |
| BsaI-K8FB-sfGFP-R | ggtctcctttacttgtacagctcgtccataccgtg |
|  |  |
|  |  |
| **Table S3**  The Codon-optimized gene sequences involved in this study. | |
| gene | sequence |
| > *RcLUS*  from *Ricinus communis* | atgtggcgaatcaagatcgccgagggcggaaacaacccctacatctactctaccaacaacttccagggccgacagatctgggtgtttgaccccaatgccggaactcccgaagaacaagccgaagtggaagaagccagacagaacttctggaagaaccgattccaggtgaagcccaactctgacctgctgtggcagctgcaattcctgcgagagaagaacttcaagcagaagatccccaaggtgaaggtggaagacggcgaggaaatcacctctgagattgctgctgccgctctgagaagatctgtgcatctgttctctgctctgcaggcctctgatggacattggtgcgctgaaaatggcggcctgctgttttttcttccccccctggtgtttgctgtgtacatcaccggacacctgaacaccgtgttctctcccgagcaccgaaaggagatcctgcgatacatctactgccaccagaatgaagatggcggctggggaattcatatcgagggccactctaccatgttctgcaccgtgctgaactacatctgcatgcgaatcctgggcgaagctagagatggcggaattgagaacgcttgtgagcgaggcagaaagtggattctggaccatggaggagccaccggaatttcttcttggggcaagacctggctttctatcctgggcgtgtacgaatgggacggaaccaaccctatgcctcccgagttttgggctttcccctcttctttccctctgcaccccgctaagatgttctgctactgccgaatcacctacatgcccatgtcttacctgtacggcaagagattcgtgggccccatcactcctctgatcctgcaaatccgagaggagatctacaacgagccctacaacaagatcaagtggaactctgtgcgacacctgtgcgctaaggaggacaactatttcccccaccccaccatccaaaagctgctgtgggatgccctgtacaccttttctgagcccctgttctctcgatggcccttcaacaagctgcgagagaaggccctgaagatcaccatggaccacatccactacgaggaccacaactctcgatacatcaccatcggctgcgtggaaaagcccctgtgcatgctggcttgttggatcgaggacccccatggcgaggctttcaaaaagcacctggcccgaatcgccgactatatctgggttggagaggacggcatcaagatgcagtctttcggctctcagacctgggatacctctctggctctgcaagccctgattgcctctgatctgtctcacgagatcggacccactctgaaacagggccacgtgttcaccaagaactctcaggccaccgagaatccttctggcgacttccgaaagatgttccgacacatctctaagggcgcctggaccttctctgataaggaccagggctggcaagtgtctgattgcaccgccgagtctctgaaatgctgcctgctgttctctatgatgccccccgagatcgtgggagaaaagatggagcccgagaaggtgtacgactctgtgaacgtgatcctgtctctgcagtctcagaacggaggattcacagcctgggaacctgctagagctggctcttggatggaatggctgaaccccgtggagttcatggaagacctggtggtggagcacgagtatgtggagtgtacctcttctgccatccaagccctggtgctgttcaagaagctgtacccccgacaccgaaacaaggagatcgagaactgcatcatcaacgccgcccagttcatcgagaacatccaggagcctgacggctcttggtatggcaattggggcatctgcttctcttacggcacctggtttgcccttaaaggcctggctgctgctggaagaacctacgagaactgctctgccatccgaaagggcgttgacttcctgctgaagtctcagagagatgatggcggctgggctgaatcttacctgtcttgccccaagaaggtgtacgtgcccttcgagggcaaccgatctaacctggtgcagaccgcttgggctatgatgggactgatctacggcggacaggccaaaagagatcccatgcccctgcatagagctgccaagctgctgatcaactctcagaccgacctgggcgatttccctcagcaagagctgactggcgctttcatgcgaaactgcatgctgcactacgccctgttccgaaacaccttccccatttgggctctggccgaatatcgacgacacgtgctgtttccttctgccggattcggattcggcttcaccaacaacctgtaa |
| > *BPLO*  from *Betula platyphylla* | atggagcacttctacctgtctctgctgctgctgttcgtgtctttcgtgaccctgtctctgttcaccctgttctacaagcaccgatctcacttcaccggccctaatctgccccctggaaagaccggatatcccatgatcggcgagtctctggagtttctgtctaccggctggaaaggacaccccgagaagttcatcttcgaccgaatgaccaagttctcttctgaggtgttcaagacctctctgctgggacaacccgctgctgtgttttgtggagccgcctgtaacaagttcctgttctctaacgagaacaagctggtgactgcttggtggcccgactctgtgaacaagatcttcccctcttctacccagacctctaactctaaggaggaggccaagaagatgcgaaagctgctgccccagttccttaaacctgaggccctgcagaagtacatctctatcatggacaccatcgcccaacgacacttcgcttctggatgggagggacagaaagaggtgactgtgttccccctggctaagcgatacaccttctggctggcctgtcgactgttcctgtctctggaggaccccaaccacatcgctcgatttgccgaccctttccactctgtggcctctggcatcatctctatccccatcgacctgcctggcactccttttaaccgaggcatcaaggcctctaacttcatccgaaaggagctgtctctgatcatcaagcagcgacgagtggatctgggcgaaggaaaagcttctcccacccaggatatcctgtctcacatgctgctgacctctgacgagtctggccagtacatgaccgaactggacatcgccgacaagattctgggcctgctgattggaggccacgatactgcttctgctgcctgcactttcatcgtgaagtacctggccgagctgccccatatttatgagggcgtgtacaacgagcagatggagatcgccaactctaaagcccctggcgagctgttaaattgggaggacatccagaagatgcgatactcttggaacgtggcctgtgaggtgctgagactggctcctcctctgcagggagcttttagagaggccatcaacgacttcatcttcaacggcttctctatccccaagggctggaagctttactggtctgccaactctacccaccgatctgccgagtatttccccgagcccgagaaatttgacccctctcgattcgaaggaagaggccccgctccctatacctttgtgccctttggcggaggccctagaatgtgtcccggcaaagagtacgccagactggagatcctggtgttcatgcacaacctggtgaagcgattccgatgggagaagatgatccccgacgagaagatcgtggtggaccctatgcccatgcctgctaagggactgcctgtgcgactgtaccctcataaggcctaa |
| > *CrAO*  from *Catharanthus roseus* | atggagatcttctacgtgaccctgctgtctctgttcgtgctgctggtgtctctgtctttccacttcctgttctacaagaacaagtctaccctgcccggacctttacctcctggaagaactggctggcctatggtgggagagtctctgcagtttctgtctgccggctggaaaggacatcccgagaagttcatcttcgaccgaatggccaagtactcttctaacgtgttccgatctcacctgctgggagaacctgctgctgtgttctgtggagctatcggcaacaagttcctgttctctaacgagaacaagctggtgcaagcttggtggcccgactctgtgaacaaggtgttcccctcttctaaccagacctcttctaaggaggaggccatcaagatgcgaaagatgctgcccaacttcctgaaacctgaggccctgcagagatacatcggcctgatggaccagattgcccagaagcacttctcttctggctgggagaaccgagaacaggtggaagtgttccccctggccaagaactacaccttctggctggcctctcgactgttcgtgtctgtggaggaccctatcgaagtggccaagctgctggagcctttcaacgtgcttgcctctggcctgatttctgtgcctatcgaccttcccggcactccttttaaccgagccatcaaggcctctaaccaggtgcgaaagatgctgatctctatcatcaagcagcgaaagatcgatctggctgagggcaaagcttctcccacccaggatatcctgtctcacatgctgctgacctctgacgagaacggcaagttcatgcacgagctggacatcgccgataagatcctgggcctgctgattggaggacacgacaccgcttcttctgcctgcaccttcatcgtgaagttcctgggcgagctgcccgaaatttacgagggcgtgtacaaggagcagatggagatcgccaactctaaagcccccggcgaatttcttaactgggaggacatccagaagatgaagtactcttggaacgtggcctgtgaggtgcttagactggctcctcctctgcaaggagctttcagagaggccctgaacgacttcatgttccacggcttctctatccccaagggctggaagatctactggtctgtgaactctacccaccgaaaccccgagtgttttcccgatcccctgaagtttgacccctctcgattcgatggatctggacccgctccctacacttttgtgcccttcggaggaggccctagaatgtgtcccggaaaggagtacgctcgactggagatcctggtgttcatgcacaacctggtgaagcgattcaagtgggagaagatcatccccaacgagaagatcgtggtggaccccatgcccatccctgaaaaaggcctgcctgtgagactttacccccacatcaatgcctaa |
| > *MtAO12*  from *Medicago truncatula* | atggagcccaacttctacctgtctctgctgctgctgttcgtgtctttcatctctctgtctctgttcttcatcttctacaagcagaagtctcccctgaatctgcctcctggcaaaatgggataccccatcatcggcgagtctctggagttcctgtctaccggctggaaaggacaccccgagaagttcatcttcgaccgaatgcgaaagtactcttctgagctgttcaagacctctatcgtgggcgagtctaccgtggtttgttgcggagccgcttctaacaagttcctgttctctaacgagaacaagctggtgactgcctggtggcctgactctgtgaacaagatcttccccaccacctctctggactctaacctgaaggaggagtctatcaagatgcgaaagctgctgccccagttctttaagcctgaggccctgcagagatacgtgggagtgatggacgtgattgcccagcgacacttcgttacccactgggacaacaagaacgagatcaccgtgtaccccctggctaagcgatacaccttcctgcttgcctgccgactgttcatgtctgtggaggacgagaaccacgtggccaagttctctgaccccttccaacttattgccgccggcattatctctctgcccatcgatctgcctggaaccccctttaacaaggccatcaaggcctctaacttcatccgaaaggagctgatcaagatcatcaagcagcgacgaatcgacctggctgaaggaactgcttctcccacccaggatatcctgtctcacatgctgctgacctctgacgagaacggcaagtctatgaacgagctgaacatcgccgacaagatcctgggcctgctgattggaggacacgacaccgcttctgtggcttgcaccttcctggtgaagtacctgggagaactgccccacatctacgacaaggtgtaccaggagcagatggagatcgccaagtctaagcccgctggagagctgctgaattgggacgacctgaagaagatgaagtactcttggaacgtggcctgcgaggtgatgagactttctccccccctgcaaggaggatttcgagaggccatcaccgacttcatgttcaacggcttctctatccccaagggctggaagctgtactggtctgccaactctacccacaagaacgccgagtgtttccccatgcccgagaaatttgaccccacccgatttgaaggaaatggccctgccccttatacctttgtgcctttcggaggcggacctagaatgtgtcccggaaaggagtatgctcgactggagatcctggtgttcatgcacaacctggtgaagcgattcaagtgggagaaggtgatccccgacgagaagatcatcgtggaccccttccccatccctgctaaggatctgcccatccgactgtatccccacaaagcctaa |
| > *VvAO15*  from *Vitis vinifera* | atggaggtgttcttcctgtctctgctgctgatcttcgtgctgtctgtgtctatcggcctgcacctgctgttctacaagcaccgatctcacttcaccggacctaatctgccccccggaaaaattggatggcccatggtgggagagtctctggagttcctgtctactggctggaagggccatcctgagaagttcatcttcgaccgaatctctaagtactcttctgaggtgttcaagacctctctgctgggcgaacctgctgctgtgtttgctggagctgccggaaataagttcctgttctccaacgagaacaagctggtgcatgcctggtggccttcttctgtggacaaggtgttcccctcttctacccagacctcttctaaggaggaggccaagaagatgcgaaagctgctgccccagttctttaaacccgaggccctgcagagatacatcggcatcatggaccacattgcccagcgacactttgctgactcttgggacaaccgagatgaggtgatcgtttttcccctggccaagcgattcactttctggctggcctgccgactgttcatgtctatcgaggaccctgcccacgtggctaaattcgagaagcccttccacgtgctggcttctggactgatcaccgtgcctattgatcttcccggcaccccttttcatagagccatcaaggcctctaacttcatccgaaaggagctgcgagccatcatcaagcagcgaaagatcgacctggctgagggcaaggcttctcagaaccaggacatcctgtctcacatgctgctggccactgacgaagacggatgccacatgaacgagatggagatcgccgacaagatcctgggacttctgatcggcggccatgatactgcctctgccgctatcaccttcctgatcaagtacatggccgagctgccccacatttacgagaaggtgtacgaggagcagatggagatcgccaactctaaagcccctggcgaactgcttaattgggacgacgtgcagaacatgcgatactcttggaacgtggcctgcgaggttatgagactggctccccctctgcaaggagctttcagagaagccatcaccgacttcgtgttcaacggcttctctatccccaagggctggaagctgtactggtctgccaactctacccacaagtctcccgagtgcttccctcagcccgaaaacttcgatcccacccgatttgaaggcaatggccccgctccttataccttcgtgccctttggaggcggacctagaatgtgtcccggcaaggagtatgccagactggagatcctggtgttcatgcacaacgtggtgaagcgattcaagtgggacaagctgctgcccgacgagaagattatcgtggaccccatgcctatgcctgctaaaggactgcccgtgagactgcaccctcacaaaccttaa |
| > *VvAO17*  from *Vitis vinifera* | atggaggtgttcttcctgtctctgctgctgatctctgtgctgtctgtgtctatccgactgtacctgctgctgtacaagcaccgatctcacttcaccggccctaatctgccccccggcaaaattggatggcccatggtgggagagtctctggagttcctgtctaccggctggaaaggacaccccgagaagttcatcttcgaccgaatctctaagtactcttctgaggtgttcaagacctctctgctgggagaacccgctgctgtttttgctggagccgccggcaataaattcctgttctccaacgagaacaagctggtgcatgcctggtggccttcttctgtggacaaggtgttcccctcttctacccagacctcttctaaggaggaggccaagaagatgcgaaagctgctgccccagttcctgaaacctgaggctctgcaacgatacaccggcatcatggaccatatcgcccagcgacacttcgctgactcttgggacaatcgagatgaggtgatcgtgttccctctggccaagcgattcaccttttggctggcctgccgactgttcatgtctatcgaggaccctgcccatgtggccaaattcgagaagcccttccacgtgcttgcttctggcctgatcaccatccctattgacctgcccggaaccccttttcatcgagctatcaaggcctctaacttcatccgaaaggagctgcgagccatcatcaagcagcgaaagatcgacctggccgagtctaaggcctctaagacccaggacatcctgtctcacatgctgctggccaccgatgaagatggctgccacatgaacgagatgtctatcgccgacaagatcctgggcctgctgatcggaggacacgataccgcttcttctgccatcaccttcctggtgaagtacatggctgagctgccccacatttacgagaaggtgtacaaggagcagatggagatcgccaactctaaggctcctggcgagctgctgaattgggacgacgtgcagaagatgcgatactcttggaacgtggcctgcgaagtgatgcgactggctcctcctctgcaaggcgcttttcgagaggccattaccgacttcgtgttcaacggcttctctatccccaagggctggaagctttactggtctgccaactctacccacaagtctctggagtgcttcccccagcctgagaaattcgaccccacccgattcgaaggagctggacctgccccttataccttcgtgccctttggcggaggccctagaatgtgtcctggcaaggagtacgctcgactggagatcctgatcttcatgcacaacctggtgaagcgattcaagtgggacaagctgctgcccgacgagaagattatcgtggaccccatgcctatgcctgctaagggactgcctgtgagactgcatccccacaaaccttaa |
| > *ATR2*  from *Arabidopsis thaliana* | atgaagaacatgatgaactacaagctgaagctgtgttctgtgtccaagaactctaagggagtctctctgtcccccacccctcacctgaccaagcctcccaccatccacaccgagcgagatctgctgctgccctcttcctctttcttcttcctgctgctgtcctcttcctcttacaacatctacaacgccatgtcctcttcctcttcctcttccacctctatgatcgacctgatggccgctatcattaagggagagcccgtgattgtctccgaccccgccaacgcttctgcctacgagtccgtggccgctgagctgtcttccatgctgatcgagaaccgacagttcgctatgatcgtgaccacctctattgccgtcctgatcggctgcattgtgatgctggtctggcgacgatctggctccggaaactccaagcgagtggagcccctgaagcccctggtcatcaagccccgagaggaagagattgacgacggtcgaaagaaggtgaccatcttcttcggcacccagaccggaaccgctgagggtttcgctaaggctctgggagaggaagccaaggcccgatacgagaagacccgattcaagattgtggacctggacgactacgccgctgacgacgacgagtacgaggagaagctgaagaaggaagacgtcgccttcttcttcctggctacctacggtgacggcgagcctaccgacaacgctgctcgattctacaagtggttcaccgagggcaacgaccgaggagagtggctgaagaacctgaagtacggcgtgttcggactgggtaaccgacagtacgagcacttcaacaaggtggctaaggtggtcgacgacatcctggtcgagcagggagctcagcgactggtgcaagtgggcctgggcgacgacgaccagtgtatcgaggacgacttcaccgcttggcgagaggctctgtggcctgagctggacaccattctgcgagaggaaggcgacaccgctgtggctaccccttacaccgctgctgtgctggagtaccgagtctctatccacgactccgaggacgctaagttcaacgacattaacatggccaacggtaacggctacaccgtcttcgacgcccagcacccctacaaggctaacgtggccgtcaagcgagagctgcacacccccgagtctgaccgatcctgtatccacctggagttcgacattgccggatctggtctgacctacgagactggtgaccacgtgggcgtcctgtgcgacaacctgtccgagactgtggacgaggccctgcgactgctggacatgtctcccgacacctacttctccctgcacgccgagaaggaagacggaacccccatttcttcttctctgccccctcccttccctccctgtaacctgcgaaccgctctgacccgatacgcctgcctgctgtcctctcccaagaagtctgctctggtggctctggctgctcacgcttctgaccctaccgaggctgagcgactgaagcacctggcttctcccgccggcaaggacgagtactccaagtgggtggtcgagtctcagcgatccctgctggaggtcatggctgagttcccctctgccaagcctcccctgggtgtgttcttcgctggagtggctccccgactgcagcctcgattctactccatctcctcttcccccaagatcgctgagactcgaattcacgtgacctgcgccctggtctacgagaagatgcccaccggacgaattcacaagggtgtgtgttctacctggatgaagaacgctgtcccctacgagaagtccgagaactgctcttccgcccccatcttcgtgcgacagtctaacttcaagctgccctctgactccaaggtccccatcattatgattggtcctggaaccggtctggctcccttccgaggattcctgcaggagcgactggccctggtggagtctggagtcgagctgggtccctccgtgctgttcttcggttgtcgaaaccgacgaatggacttcatctacgaggaagagctgcagcgattcgtggagtctggagctctggctgagctgtctgtcgccttctcccgagagggacccaccaaggagtacgtccagcacaagatgatggacaaggcttctgacatctggaacatgatttcccagggagcctacctgtacgtgtgcggcgacgctaagggaatggcccgagatgtgcaccgatctctgcacaccattgctcaggagcagggctctatggactccaccaaggccgagggattcgtgaagaacctgcagacctccggtcgatacctgcgagatgtgtggtaa |
| > *CrCPR*  from *Catharanthus roseus* | atggactcttcttctgagaagctgtctcccttcgagctgatgtctgccattctgaagggcgctaagctggacggctctaactcttctgactctggcgtggccgtttctcctgctgtgatggctatgctgctggagaacaaggagctggtgatgatcctgaccacctctgtggctgtgctgattggatgcgtggtggtgctgatctggcgacgatcttctggctctggcaagaaagttgtggagccccccaaactgatcgtgcccaagtctgtggtggaacccgaggaaatcgacgagggcaagaagaagttcaccatcttcttcggcacccagactggaactgctgagggattcgctaaagccctggccgaggaagctaaagcccgatacgagaaggccgtgatcaaggtgatcgacatcgacgactacgctgccgatgacgaggaatacgaggagaagttccgaaaggagactctggccttctttatcctggccacctatggcgatggcgaacctactgataatgccgcccgattctacaagtggttcgtggagggcaatgatagaggcgactggctgaagaatctgcagtacggcgtgttcggcctgggaaatcgacagtacgagcacttcaacaagatcgccaaggtggtggacgaaaaggtggctgagcagggaggaaaacgaatcgtgcctctggtgctgggagatgacgaccaatgcatcgaggacgactttgccgcctggcgagaaaatgtgtggcccgagctggataatctgctgcgagatgaggatgacaccaccgtgtctactacttacaccgccgccatccccgaatacagagtggtgttccccgacaagtctgactctctgatctctgaggccaacggacacgctaatggctacgccaatggcaacaccgtgtacgatgcccagcatccctgcagatctaatgtggccgtgcgaaaggaacttcatacccccgcctctgatagatcttgcacccacctggactttgatatcgccggcaccggactttcttatggcactggcgaccatgtgggagtgtactgcgacaacctgtctgaaaccgtggaagaggctgaacgactgctgaatctgcctcccgaaacctacttttctctgcacgccgacaaagaagatggaacccccctggctggatcttctctgccccctccttttcctccttgcactctgagaactgccctgaccagatatgccgacctgctgaacacccccaagaagtctgccctgctggctctggctgcttatgcttctgaccccaacgaagctgaccgactgaagtacctggcttctcctgccggcaaggatgaatacgcccagtctctggtggccaaccagagatctctgctggaggtgatggccgaattcccctctgctaagcctcccctgggagtgttttttgccgccatcgctcctagactgcaaccccgattctactctatctcttcttctccccgaatggccccttctcgaatccatgtgacttgcgccctggtgtatgagaaaacccccggaggccgaattcataaaggcgtgtgctctacctggatgaagaacgccatccccctggaagaatctcgagactgctcttgggctcccatcttcgtgcgacagtctaacttcaagctgcccgccgatcctaaagtgcccgtgatcatgattggacccggaactggactggctccctttagaggcttcctgcaggaacgactggctctgaaggaggagggagctgaactgggcacagccgtgtttttcttcggctgccgaaaccgaaagatggactacatctacgaggacgagctgaaccacttcctggagatcggcgctctgtctgaacttctggtggccttctctagagaaggccccaccaagcaatatgtgcagcacaagatggccgagaaggcctctgacatctggcgaatgatctctgacggcgcctacgtgtatgtgtgtggcgatgccaaaggcatggccagagatgtgcatcgaaccctgcatactatcgctcaggagcagggctctatggactctactcaggccgagggcttcgttaagaacctgcagatgaccggcagatacctgcgagatgtgtggtaa |
| > *GuCPR*  from *Glycyrrhiza uralensis* | atgacctccaactctgacctggtccgaaccatcgagtctgtcctgggtgtgtccctgggcgactctgtgtctgactccctggtgctgattgctaccacctctgtggccgtcatcattggactgctggtgttcctgtggaagaagtcttccgaccgatctcgagaggtccgacccgtgatcgtccccaagtccctggtgaaggacgaggacgacgacgtggacgtcgcttccggcaagaccaaggtgaccgtcttcttcggaacccagaccggcaccgctgagggtttcgctaaggctctggccgacgagattaaggctcgatacgagaaggcctacgtgaaggtggtcgacctggacgactacgccatggacgacgaccagtacgaggagaagctgaagaaggagactctggctttcttcatgctggctacctacggcgacggagagcctaccgacaacgctgctcgattctacaagtggttcaccgagggcaaggaagagcgaggaacctggctgcagcagctgacccacggagtcttcggtctgggcaacaagcagtacgagcacttcaacaagatcggaaaggtggtcgacgaggacctgtctgagcagggtgctaagcgactggtgcccctgggactgggtgacgacgaccagtccattgaggacgacttctctgcctggaaggagtctctgtggcctgagctggaccagctgctgcgagatgaggacgacgtcaacaccgtgtctaccccctacaccgccgctatccccgagtaccgagtggtcattcacgactctaccgtcaccccctcctacgacaaccagttctccgccgctaacggcggagctgtgttcgacatccaccacccctgtcgagtgaacgtcgccgtgaagcgagagctgcacaagccccagtctgaccgatcctgcattcacctggagttcgacatctctggcaccggaattacctacgagactggcgaccacgtcggtgtgtacgctgagaactgtgacgagactgtggaggaagccggcaagctgctgggacagaacctggacctgctgttctccctgcacaccgacaacgaggacggcacctctctgggtggctccctgctgcctcccttccccggaccttgcaccctgcgaaccgctctggctcgatacgctgacctgctgaaccctccccgaaaggctgctctggtggtcctggctgctcacgcttctgagccttctgaggccgagcgactgaagttcctgtcttctccccagggcaaggacgagtactctaagtgggtggtcggctctcagcgatccctgctggaggtcatggctgagttcccctccgccaagcctcccctgggagtgttcttcgctgctattgctccccgactgcagcctcgatactactctatttcttcctctccccgattcgcttcccagcgagtccacgtgacctgtgctctggtctacggtcctacccctaccggtcgaatccacaagggagtgtgttctacctggatgaagaacgctattcccctggaggagtcccgagactgtggttgggctcccatcttcattcgaccctctaacttcaagctgcccgccgaccactccatccccatcattatggtgggtcctggcaccggactggctcccttccgaggattcctgcaggagcgattcgccctgaaggaagacggtgtgcagctgggcccctctctgctgttcttcggttgtcgaaaccgacagatggacttcatctacgaggacgagctgaagaacttcgtcgagcagggttctctgtccgagctgattgtggccttctcccgagagggccccgagaaggagtacgtgcagcacaagatgatggacaaggccgcttacctgtggtccctgatctcccagggaggttacctgtacgtctgtggcgacgccaagggtatggctcgagatgtgcaccgaatcctgcacaccattgtccagcagcaggagaacgtggagtcctctaaggctgaggccattgtcaagaagctgcagatggacggccgatacctgcgagatgtgtggtaa |
| > *LjCPR*  from *Lotus japonicus* | atggaggagtcttcttctatgaagatctctcccctggacctgatgtctgccatgatcaagggcactctggacccctctaacgtgtcttctacctctggcgccggatctgtgtttctggagaaccgagagttcgtgatggtgctgaccacctctatcgctgtgctgatcggatgcgtggtggtgtttatctggcgacgatctaccggcaacaaggccaagtctatcgagccccccaaacgagtggtggagaagctgtctgacgaggccgaagtggatgacggaacccgaaaggtgaccatcttcttcggcacccagactggaactgctgagggcttcgccaaagccattgccgaagaggccaaggtgagatacgagaaggccaagttcaagatcgtggacatggacgactacgcccaggacgacgatgagtacgaggagaagctgaagaaggaaaccctggccctgttttttctggccacctacggagatggagaacccaccgataatgccgcccgattctacaagtggttcctggagggcgatgagaaggaagaaggctggctgcgaaaccttgagtatgccgtgttcggcctgggaaacagacagtacgagcacttcaacaaggtggccatcgaggtggatgataagctggccgacttcggcggaaaacgactggtgaaggtgggactgggtgacgatgaccagtgtatcgaggacgacttcaccgcctggaaagaggaactgtggcctgctctggacgaactgctgagaggtgacgatgacaccactgtgtctaccccctacacagctgccgtgctggaataccgagtggtgattcacgaccctctggacgcttctgtggacgagaagaagtggcacaacgtgaacggccacgctatcgtggatgctcaacaccccgtgcgatctaatgtggccgtgcgaaaggagttacacacccctgtgtctgaccgatcttgcacccacctggagttcgacatctctggaaccggcgtggcttatgaaaccggagatcatgtgggagtgtactgcgagaacctgtctgagactgtggaggaagctgtgagactgctgggactgtctcctgacacctacttctctgtccacaccgacgacgaagacggaaaacccctgtctggatcttctctgccccccacttttcctccttgcaccctgagaactgctatcgctagatacgccgacgtgctgtcttctcccaagaagtctgtgctgctggctcttgccgctcatgcttctaatccctctgaggctgacagactgcgacatctggcttctcctgccggcaaggacgaatactctgagtgggtgatcgcctctcagcgatctctgctggaggtgatggccgagttcccttctgctaagccccccatcggagtgttcttcgctgctattgcccccagactgcaaccccgattctactctatctcttcttctccccgaatggccccttctcgaatccatgtgacctgcgccctggtgaacgacaaaatgcccaccggcagaattcatcgaggcgtgtgctctacctggatgaagaactctgtgcccctggagaagtctcaggactgctcttgggctcccatcttcgtgcgacagtctaacttcaagctgcccgccgacaacaaagtgcccatcatcatgatcggacccggaactggacttgctccctttcgaggattcctgcaagaacgactggccctgaaggaagatggagctgagctgggaccttctgtgctgtttttcggctgccgaaaccgacagatggactacatctacgaggacgagctgaaccacttcgtgaactctggcgccctgtctgaacttatcgtggccttctctagagaaggccccaccaaggaatacgtgcagcacaagatgatggagaaggcctctgacatctggaacatgatctctcagggcgcctacatttatgtgtgcggcgacgccaaaggaatggctcgggacgtgcatcgaactctgcacaccatcctgcaagagcagggctctctggactcttctaaggccgagggcatggtgaaaaacctgcagctgaacggccgatacctgcgagatgtgtggtaa |
| > *MTR2*  from *Medicago truncatula* | atgacctcttccaactccgacctggtccgaaccatcgagtctgtgctgggtgtctccctgggcgactccgtgtctgactccgtggtcctgattgtgaccacctctgccgctgtcatcattggactgctggtgttcctgtggaagaagtcttccgaccgatccaaggagctgaagcccgtgatcgtccccaagtctctggtgaaggaagaggacgacgacgctgacattgccgacggcaagaccaaggtgaccgtcttcttcggaacccagaccggcaccgctgagggtttcgctaaggctctggccgaggagatcaaggctcgatacgagaaggccttcgtgaaggtggtcgacatggacgactacgccgctgacgacgaccagtacgaggagaagctgaagaaggagactctggctttcttcatgctggctacctacggcgacggagagcctaccgacaacgctgctcgattctacaagtggttcaccgagggcaaggacgagcgaggaacctggctgcagcagctgacctacggagtcttcggtctgggcaaccgacagtacgagcacttcaacaagatcggaaaggtggtcgacgacgacctgtctgagcagggtgctaagcgactggtgcccctgggaatgggtgacgacgaccagtccattgaggacgacttcaacgcttggaaggagtctctgtggcccgagctggaccagctgctgcgagatgaggacgacgtgaacaccgtgtctaccccctacaccgccgctatttccgagtaccgagtggtcttccacgaccccaccgtcaccccctcttacgagaaccacttcaacgccgctaacggcggagccgtgttcgacatccaccacccctgtcgagctaacgtggccgtccgacgagagctgcacaagccccagtctgaccgatcctgcattcacctggagttcgacgtgtccggcaccggagtcacctacgagactggcgaccacgtgggtgtctacgctgacaactgtgacgagactgtcaaggaagccggcaagctgctgggacaggacctggacctgctgttctctctgcacaccgacaacgaggacggcacctctctgggtggctccctgctgcctcccttccccggaccttgcaccgtgcgaaccgctctggctcgatacgctgacctgctgaaccctccccgaaaggctgctctgattgctctggctgctcacgcttctgagccttctgaggccgagcgactgaagttcctgtcttctccccagggcaaggacgagtactctaagtgggtggtgggctcccaccgaaccctgctggaggtcatggctgacttcccctccgccaagcctcccctgggagtgttcttcgctgctattgctccccgactgcagcctcgatactactctatttcttcctctccccgattcgctccccagcgagtgcacgtcacctgtgctctggtggagggtcctacccctaccggtcgaatccacaagggagtgtgttccacctggatgaagaacgccattccctctgaggagtcccgagactgctcttgggctcccatcttcattcgaccctccaacttcaagctgcccgccgacccctctattcccatcattatggtgggtcctggcaccggactggctcccttccgaggattcctgcaggagcgattcgccctgaaggaagacggtgtgcagctgggtcctgctctgctgttcttcggctgtcgaaaccgacagatggacttcatctacgaggaagagctgaacaacttcgtcgagcagggttctctgtccgagctgattgtggccttctcccgagagggccccgagaaggagtacgtccagcacaagatgatggacaaggcttcttacttctggtccctgatctcccagggaggttacctgtacgtgtgtggcgacgccaagggtatggctcgagatgtgcaccgaaccctgcacaccattgtccagcagcaggagaacgccgactcctctaaggctgaggccaccgtcaagaagctgcagatggacggccgatacctgcgagatgtgtggtaa |
| > *EMC*  from *Mucor circinelloides* | atgtctcccatcattgacttcgtgcgacgacagctgtcttccaccaagctgcacgaggagcagcagaccgccaccaccaacgacctggtgtctcgatccggctacctgaacgagggaaagtacgaggtgcgactgaactgtattaacgctggatgcctgcagaagaagctgaactacatcggtaccgccatggaccccgctaagcgacagcgactgggcctgaacggactgctgcctgccggagtcgagactctggagattcagaaggctcgagccctgcgagtgctgcgatctaagcacaacctgctggagaagtacatcctgatggcccagctgcgaaccaccaacgtccgactgttctacaagatcgtgattgacgagctggagactgtccagctggctcccgtgatctacacccctaccgtcggcaccgcttgtctggagtactccaccatctaccccttcctggctgctcctggtgtgcctgacggcctgtacctgaccaaggccgagctgcccgagctctgccagaccattcgaaactaccgacctaccgacaccgagggtttcgagcctgagatcgctgtcatttctgacggatcccgaattctgggcctgggcgacctgggtaccaacggtatgggcatccccatgggaaagctgcagctgtacgtggctggagccggtattgacccccgacgaaccctgcccatcattctggacctgggaaccaacaacgagaagctgctgaacgacgagttctacatcggtctgcgacagaagcgacccaacgacgaggagttctaccagaccgtggacaccgtcctgaccgccctgcacaccgtctaccccaacctgctgatccagttcgaggactggtcttccgagcacgctttcggactgctggagaagtaccagaaccagatgctgtgtttcaacgacgacattcagggcaccggagccgtcatcctgtctggcgtgatcaacgctattcgaaaggtcgagaaggagaaccaggtgtccccccgagatcaccgaattgtcttctacggtgctggttctgctgctatcggagtggctcgacagattcagtcctacttccagatcgagcacaacatgaccgaggaggaggccaagcacgtgttctggatcgtcgactctaagggactggtcaccaccacccgaggtgacaagctggcccagcacaaggtgtactacgctcgaggagacaacgagggtcagcagtacaaggagctgatcgacattgtgaactacaacctgtactccctgattggtctgtcttccaccaccggcgccttcaacacccaggtcctggagcgactggcttctctgaacgagcagcccatcgtgttccctctgtccaaccctgctacccaggctgagtgtaccttcgagcaggctatggaggccaccaacaacaaggtcattttcgcctctggtaccgctttccccgcctacaccatcaagtccaccggagaggtgaacacccctggacagggtaacaacatgtacattttccctggcctgggactgggtgcttgcctggctaaccctgctcacttcgaccgaatgatctacgaggcctctaaggctctggccgactccctgaccgaggaggagatctctaaggcctggctgtacccctccctgaactaccgatctgtgtccgctattgtcgccgctgccgtgtgtcaggagactctgaacgagaacctggccacctctcaggctatgatgacccagtgcaagtcccacgaggacatcctggactacgtgtctgcccacatgtggtcccccgactacggtaacaacaactccaaccagcaggctggcaagctgtaa |
| > *EMT*  from *Mortierella alpina* | atggctctgtcttccctgtccaccctgcctcgatcttccgctgctcgatctttcttcctgcagaagggcaacaactcctgtctgcactcttccttctcctgttgctactctaccaagatctccattccccgacacaccctgaagccccgactgaccaactctctgcagtcccacacctctctggcccccctgttctctacccgactgtccaccccctctatcacccccaccaccacccactctattttccgatccctgtcttccgcctctgtggctatccccgaccacctgtccaacacccaccagcacaccgacaacatgaccggaaccgctacccctaccgagctgcgatctgctctgcacctgcagggtctgacccctgctaaggtcgagtccttcgagctgcagaagaagcgagctctggcccagctgcgatctaagtcttccgacattgagaagtacgtgttcctggcctggctgcgaaacaccaacgtgcgactgttctacggactggtcggtgaccagctggaggagactctgcccctgatctacacccccaccgtcggcaccgcttgccagaactactcttccatctaccccttcctggctcctcctggtcagcctgacggactgttcctgtccatcaacgacctgcccaacctgacccagatcattcagaactacaagcctttccctcaggacccttctctgaccccccagatcgccgtgattaccgacggttcccgaatcctgggactgggtgacctgggtgtgggtggaatgggtattcccgtcggcaagctgcagctgtacgtggctggtgctggaatcgaccctcgacgaaccctgcccattaccctggacctgggaaccaacaacgaggacaagctgaaggacgagttctacctgggcctgcgagagaagcgagctggagacgacaagttcttccccttcgtggacgccgtcatcgaggctctgacctccctgtaccccaagctgctgattcagttcgaggacttctcttccgagcacgccttccagctgctggacaagtaccagcccaagaagttctgtttcaacgacgacatccagggtaccggcgccgtcattctggctggcttcatgaacgccatcaacctggctggaattcccgccaaggaccacaagattctgttcttcggagctggttctgctgctgtgtgcgtcgctaagcagctggctgcttacttcgtcaacgaccacggtatgaccgaggacgaggctcgagagatggtgtggctggtcgactccaagggtctggtgaccctggaccgaggcgacaagctggctgagcacaagctgtacttcgctcgaaaggacaacgccggatctcagtacccctccatcgcttctgtgattgagcacgtccgacccaccgccctgttcggactgtcttcccagtctggtgctttctccgaggacgtcctgaagtccatggccaccctgaaccagcgacccatcgtgttccccctgtctaaccccgcctaccaggctgagtgttccttcgagcaggccatgatccacaccaagggcaaggtcatcttcgcttccggtaccgccttccccaagtacaccgaccccaacaccggcctgatttctgctcccggccagggaaacaacatgtacatcttccctggtctgggcctgggtggtattctggctcagcctgagactatctctgaccgactgatctactccgccgctcgagcttgcgccaacgctctgaccgtcgaggagcgatcccagggcctgctgtaccctgtgctgcctcgaatccgacaggtgtctgctgaggtcgccgctgccgtggtcaccgaggctgtcgctgagggaaccgctaccaacgaggaggccattaagctggtgaagtctcacgacaaggctgccctgctgaacttcgtcaactccaacatgtggaccaccgacggagtgcagcacgagatctctaccaccttcgccccccacctgtaa |
| > *RtMe*  from *Rhodotorula toruloides NP11* | atgcctgctcacttcgctccctctcagcctctgcagggtggaccctctccctcccagctgggacccaaggagctgctgatcgagcgagccctgacccgactgcgatccattcccaacgacctggagaagtacaccttcctggctggactgcgaggtcgaaaccccgacgtgttctacggactggtcggtggcaacatgaaggagtgttgccccatcatctacacccccgtgatcggcctggcctgtcagaactggtccctgattcaccctcctcctcctgagtccgaccctaccatcgacgctctgtacctgtcttactccgacctgcccaacctgccccagctgatcggaggtctgaagacccgactgccccacgaccagatgcagatttctgtggtcaccgacggatcccgagtgctgggcctgggcgacctgggtgtcggtggaatgggtatttctcagggcaagctgtccctgtacgtggctgctggtggtgtcaaccctaaggctaccctgcctatcgctattgacttcggcaccgacaacgagactctgctggctgaccctctgtacgtgggtcagcgaatccgacgactgtctcaggagaagtgtctggagttcatggaggtgttcatgcgatgcatgcacgagactttccccaacatggtcattcagcacgaggactggcagacccctctggctttccctctgctgcacaagaaccgagatctgtacccctgtttcaacgacgacatccagggcaccggtgctgtggtcctggctggtgctattcgagccttccacctgaacggagtcgctctgaaggaccagaagatcctgttcttcggagctggttcttccggtgtgggagtcgctgagactatttgcaagtacttcgagctgcagggaatgtctgaggacgaggccaagtccaagttctggctggtggactccaagggactggtcgctcacaaccgaggtgacaccctgccctctcacaagaagtacctggctcgatccgagcctgacgctcctaagctgcgaaccctgaaggaggtggtcgagcacgtgcagcctaccgctctgctgggcctgtctaccgtcggaggcaccttcaccaaggagatcctggaggccatggctacctacaacaagcgacccattgtgttcgctctgtccaaccctgtcgctcaggctgagtgcaccttcgaggaggctgtggagggcaccgacggacgagtcctgtacgcttctggctcccccttcgaccccgtggagtacaagggaaagcgatacgagcccggtcagggcaacaacatgtacatcttccccggactgggtatcggcgccattctggctcgagtgtctaagattcccgaggagctggtccacgcctctgctcagggactggctgactccctgacccctgaggagactgctcgacacctgctgtaccccgacatcgagcgaattcgagaggtgtccatcaagattgccgtgaccgtcatccaggccgctcagaagctgggtgtcgaccgaaacgaggagctgcggggcaagtcttccgccgagattgaggcttacgtgcgaaagggcatgtaccaccccctgctggaggccgagcagcaggctcagtaa |
| > *GaPC*  from *Kluyveromyces lactis* | atgcccgatatgaccaacgagtcctcttcgaagcccgcccagatcaacatcggcatcaacggcttcggccgaatcggacgactggtgctgcgagccgccctgacccaccccgaggtgaaggtgcgactgatcaacaacccctctaccacccccgagtacgccgcctacctgttcaagtacgactctacccacggcaagtaccgaggcgaggtcgagttcgacgacgagcgaatcatcatccagaacgaccacgtgtctgcccacatccccctgtctcacttccgagagcccgagcgaatcccctgggcctcttacaacgtggactacgtgatcgactctaccggcgtgttcaaggaagtggacaccgcctctcgacacaagggcgtgaagaaggtgatcatcaccgccccctctaagaccgcccccatgtacgtgtacggcgtgaaccacgtgaagtacaaccccctgaccgaccacgtggtgtctaacgcctcttgcaccaccaactgcctggcccccctggtgaaggccctggacgacgagttcggcatcgaagaggccctgatgaccaccatccacgccaccaccgcctctcagaagactgtcgacggcacctcttctggcggcaaggactggcgaggcggccgatcttgccagggcaacatcatcccctcttctaccggcgctgccaaggccgtgggcaagatcctgcccgagctgaacggcaagatcaccggcatgtctatccgagtgcccaccatcaacatctccctggtggacctgaccttccgaaccgccaagaagacctcttacgacgacatcatgaaggccctcgagcagcgatctcgatctgacatgaagggcgtcctgggcgtgaccaaggacgccgtggtgtcctctgacttcacctctgactctcgatcttctatcgtggacgccaaggccggcatcgagctgaacgaccacttcttcaaggtgctgtcttggtacgacaacgagtacggctactcttctcgagtggtcgacctgtctatcttcatggcccagaaggacttcgaggccggcgtgtaa |
| > *sfGFP* | atgcgaaagggtgaggagctgttcaccggtgtggtgcccatcctggtggagctggacggcgacgtcaacggtcacaagttctctgtgcgaggtgagggcgagggcgacgccactaacggtaaactgaccctgaagttcatttgtaccaccggtaaactgcccgtgccctggcccaccctggtcactaccctgacctacggcgtgcagtgtttcgcccgataccccgaccacatgaagcagcacgacttcttcaagtccgccatgcccgagggctacgtgcaggagcgaaccatctccttcaaggacgacggcacctacaagacccgagccgaggtgaagttcgagggtgacaccctggtgaaccgaatcgagctgaagggtatcgacttcaaggaggacggtaacatcctgggccacaagctggagtacaacttcaactctcacaacgtctacatcaccgccgacaagcagaagaacggcatcaaggccaacttcaagatccgacacaacgtcgaggacggctccgtgcagctggccgaccactaccagcagaacacccccatcggtgacggtcccgtgctgctgcccgacaaccactacctgtccacccagtccgtcctgtctaaggaccccaacgagaagcgagatcatatggtgctgctggagttcgtgaccgccgccggcatcacccacggtatggacgagctgtacaagtaa |
